# Breast Cancer Stem Cell-Associated Extracellular Vesicles: Bioengineering Advances in Source-Resolved Isolation, Mechanistic Validation, and Clinical Translation

**DOI:** 10.3390/bioengineering13101196

**Published:** 2026-10-14

**Authors:** Nuan Yang, Yuan Gao, Weijie Li, Yuancong Jiang, Yingjie Wang, Liwei Meng, Cheng Chen

**Affiliations:** 1 School of Medicine, Shaoxing University, Shaoxing 312000, China; yn19883581236@usx.edu.cn (N.Y.);; 2 Department of Breast and Thyroid Surgery, Shaoxing People’s Hospital, Shaoxing 312000, China; 3 Department of Pulmonary and Critical Care Medicine, Shaoxing People’s Hospital, Shaoxing 312000, China; 4 State Key Laboratory for Diagnosis and Treatment of Infectious Diseases, National Clinical Research Center for Infectious Diseases, Collaborative Innovation Center for Diagnosis and Treatment of Infectious Diseases, The First Affiliated Hospital, School of Medicine, Zhejiang University, Hangzhou 310003, China; yingjiewang@zju.edu.cn; 5 Cancer Center, Zhejiang University, Hangzhou 310003, China

**Keywords:** breast cancer stem cells, extracellular vesicles, bioengineering, microfluidics, liquid biopsy

## Abstract

Breast cancer stem cells (BCSCs) drive therapeutic resistance, recurrence, and metastasis via self-renewal, phenotypic plasticity, and adaptive tolerance to treatment and microenvironmental stress. Extracellular vesicles (EVs) mediate bidirectional intercellular communication between stem-like tumor cells and tumor, stromal, immune and vascular compartments, but their translation is blocked by critical unaddressed gaps: BCSC rarity and heterogeneity, incomplete donor-cell validation, uncertain vesicle-source attribution, and nonstandardized assays. This review outlines a unifying bioengineering framework built around source-resolved analysis to integrate BCSC enrichment, EV characterization, functional validation and translational development and evaluate current platforms for BCSC isolation (including flow cytometry, magnetic enrichment, microfluidic sorting, affinity capture, and single-cell platforms), EV purification, biosensing, single-vesicle profiling, and multimarker detection, alongside organoid and tumor-on-chip models for dissecting EV-driven resistance and metastatic niche remodeling. BCSC-associated EV signatures complement circulating tumor cells and circulating tumor DNA for noninvasive monitoring of stemness dynamics, treatment adaptation, and recurrence risk, while engineered EVs offer a unique targeted delivery route to resistant tumor states. Real-world deployment will require direct head-to-head comparison with clinically established nanocarriers for efficacy, manufacturability, biodistribution and safety. The eventual clinical translation of this field hinges on validated BCSC identity, reliable EV source attribution, standardized detection protocols, causal mechanistic validation, and scalable manufacturing.

## 1. Introduction

Breast cancer is a heterogeneous disease whose cellular composition and molecular programs vary among patients, molecular subtypes, intratumoral regions, treatment stages, and primary and metastatic sites [1]. Within this diversity, breast cancer stem cells (BCSCs) represent a functionally defined population or cellular state characterized by self-renewal, multilineage differentiation, tumor initiation, and long-term tumor propagation. Their clinical significance is closely linked to intrinsic treatment resistance and phenotypic plasticity. Pre-existing stem-like cells may survive treatment through drug efflux, enhanced DNA damage repair, redox control, anti-apoptotic signaling, metabolic adaptation, and quiescence [2]. In parallel, inflammatory signals and therapeutic stress can induce non-BCSCs to acquire stem-like properties, thereby replenishing the resistant compartment [3]. For example, IL-6-dependent feedback signaling has been implicated in the expansion of stem-like cells during trastuzumab resistance [4], while endocrine-resistant breast cancer cells can acquire ALDH-high and sphere-forming phenotypes [5]. Consequently, BCSCs should not be regarded as a fixed minor population, but as heterogeneous and dynamically regulated cellular states that may persist or emerge under selective pressure and subsequently contribute to tumor regrowth, recurrence, and metastatic progression.

Extracellular vesicles (EVs) provide a potential mechanistic link between BCSCs, non-stem tumor cells, and the surrounding microenvironment. EVs transfer proteins, nucleic acids, lipids, and metabolites among malignant and stromal compartments, thereby contributing to local and systemic intercellular communication [6]. In breast cancer, EV-mediated communication has been demonstrated between tumor cells and cancer-associated fibroblasts, adipocytes, and other microenvironmental components [7,8,9]. EVs released by BCSCs or treatment-adapted breast cancer cells may transmit signals associated with stem-like phenotypes, epithelial–mesenchymal plasticity, therapeutic resistance, and metastatic progression [10,11]. This communication is bidirectional: tumor-cell-derived EVs may remodel stromal and recipient tumor cells, whereas microenvironment-derived EVs may promote metabolic adaptation, treatment resistance, or the maintenance of stem-like tumor states [8,9]. However, the low abundance and heterogeneity of BCSCs, together with the molecular complexity of EV populations, have limited the precise characterization of BCSC-related vesicle signaling. Conventional cell-sorting and EV-isolation methods often provide insufficient resolution to distinguish vesicles associated with stem-like tumor states from those released by the broader tumor and stromal compartments. Bioengineering technologies offer opportunities to overcome these limitations through multiparameter cell sorting, microfluidic enrichment, affinity capture, single-cell and single-EV analysis, integrated biosensing, patient-derived organoids, and tumor-on-a-chip models [12,13,14]. Throughout this review, EV nomenclature follows MISEV2023, and the term exosome is reserved for settings in which endosomal origin is directly demonstrated rather than inferred from size or marker expression alone [15].

Accordingly, the distinctive perspective of this review is to connect BCSC isolation, EV characterization, functional validation, and clinical translation within a single bioengineering framework. We first evaluate conventional and emerging platforms for enriching and functionally confirming BCSCs, emphasizing that reliable donor-cell identification is a prerequisite for attributing vesicular signals to BCSCs. We then examine engineering approaches for isolating, detecting, and functionally analyzing BCSC-associated EVs and, where donor-cell identity is rigorously verified, BCSC-derived EVs using single-vesicle technologies, organoids, and tumor-on-a-chip models. Subsequently, we assess their potential in liquid biopsy and targeted drug delivery, while comparing EV-based strategies with circulating tumor cells, circulating tumor DNA, liposomes, lipid nanoparticles, and other nanocarriers. This review aims to clarify how bioengineering advances can improve BCSC isolation, EV analysis, and the development of clinically meaningful biomarkers and therapeutic strategies.

This narrative review was based on a structured literature search of PubMed, Web of Science, Scopus, and Embase. The search covered studies published from January 2000 to August 2026, using combinations of the keywords “breast cancer stem cell,” “extracellular vesicle,” “exosome,” “bioengineering,” “microfluidic,” “isolation,” “characterization,” “liquid biopsy,” and “therapeutic delivery”. Original research articles, methodological studies, and relevant reviews published in English were included. Conference abstracts, short communications, duplicate publications, and studies with insufficient methodological or experimental information were excluded. Two investigators independently screened the retrieved records, and disagreements were resolved through discussion with a third investigator. A total of 132 studies were included in the final review.

## 2. Advanced Bioengineering Platforms for BCSC Isolation and Sorting

**Figure 1:**
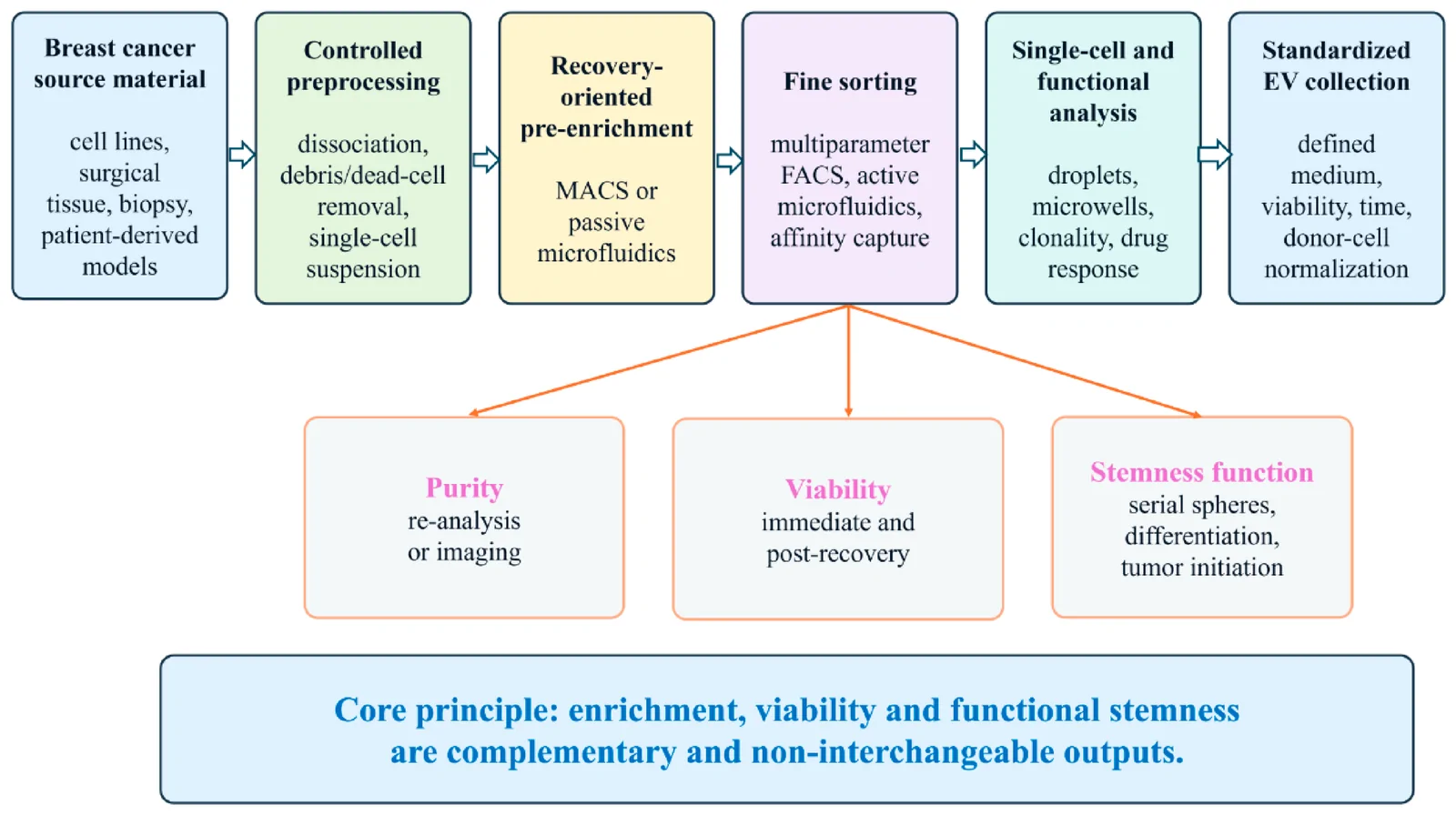
Integrated engineering workflow for BCSC isolation and validation. Breast cancer source materials, including cell lines, surgical tissues, biopsy specimens, and patient-derived models, undergo controlled preprocessing to generate viable single-cell suspensions. Recovery-oriented pre-enrichment using magnetic-activated cell sorting or passive microfluidics is followed by higher-resolution sorting using multiparameter flow cytometry, active microfluidics, or affinity-based capture. Downstream single-cell and functional analyses assess clonal behavior and treatment response, while standardized culture and collection conditions support source-resolved extracellular vesicle studies. Purity, viability, and functional stemness should be evaluated independently because these parameters are complementary but not interchangeable. EV, extracellular vesicle; FACS, fluorescence-activated cell sorting; MACS, magnetic-activated cell sorting.

Source-resolved analysis of BCSC-associated EVs critically depends on validated methods for cell isolation and stemness confirmation. Figure 1 provides an integrated bioengineering workflow that proceeds from sample preprocessing through enrichment and fine sorting to functional validation and downstream EV collection.

### 2.1. Engineering Optimization of Conventional Sorting Technologies

Conventional BCSC isolation is best organized as a staged workflow—MACS-based pre-enrichment, multiparameter FACS-based high-purity sorting, and independent functional confirmation—rather than as a set of interchangeable platforms; its reliability also depends on how the target population is defined. The identification of CD44^high^/CD24^low^ breast cancer cells with enhanced tumor-initiating capacity established an important basis for prospective BCSC isolation [16]. ALDEFLUOR-based detection subsequently identified an ALDH-high tumorigenic population that only partially overlapped with the CD44^high^/CD24^low^ fraction [17]. The relative abundance and overlap of these phenotypes differ substantially across intrinsic breast cancer subtypes, indicating that neither can serve as a universal BCSC definition [18]. Combined ALDH activity and surface-marker profiling has produced stronger enrichment in selected models, as ALDH-high/CD44-positive/CD24-negative or ALDH-high/CD133-positive populations have shown enhanced metastatic and tumor-initiating properties [19]. However, ALDH1 immunohistochemistry and the ALDEFLUOR assay should not be treated as interchangeable, because the latter measures intracellular enzymatic activity and may detect contributions from several ALDH isoforms [20]. EpCAM, CD49f, CD133, and related markers can therefore be used as supplementary recognition features, but their value must be validated within the relevant subtype, specimen, and functional context. More specifically, CD44high/CD24low enrichment is most consistently observed in basal-like and triple-negative contexts, whereas luminal tumors frequently show a lower prevalence; its distribution also diverges from that of ALDH-high populations. Tissue-based studies report that overlap between CD44/CD24-defined and ALDH1-positive populations is well below 30% and can approach 1% in some cohorts, reinforcing that no single surface-marker combination is universal across molecular subtypes [18,21].

Recent flow-cytometric protocols have enabled the identification and isolation of putative CSC or tumor-initiating populations from cultured breast cancer cells and xenograft models using marker-based gates and viability controls [22]. Engineering optimization of flow cytometry should focus on both analytical resolution and preservation of the cellular state. Multiparameter or spectral cytometry can increase marker coverage, whereas fluorescence-minus-one controls, compensation or spectral unmixing, singlet discrimination, dead-cell exclusion, and standardized gating can reduce false-positive classification. FACS has also been applied to freshly resected breast tissues using combinations such as CD49f and CD24, illustrating its ability to recover phenotypically distinct populations from clinically derived specimens [23]. In patient-derived xenograft models, sensitive fluorescence-based isolation has enabled downstream single-cell analysis of rare metastatic and stem-like populations [24]. Nevertheless, prolonged sample preparation, fluorescent substrates, antibody binding, high pressure, shear stress, and temperature changes may impair viability or alter marker expression. Low-pressure sorting, an appropriate nozzle diameter, shortened processing time, temperature-controlled collection medium, and immediate post-sort assessment are therefore important when the isolated cells will be used for culture, organoid establishment, sequencing, or EV collection [25].

MACS provides a complementary strategy when rapid enrichment, large input volumes, or removal of unwanted cells is required. Semi-automated tissue dissociation followed by magnetic enrichment has enabled transcriptomic analysis of small numbers of breast cancer stem-like cells obtained from primary tumors [26]. MACS-based enrichment of CD44-positive/CD24-low populations has also been used to generate BCSC-enriched fractions for therapeutic and mechanistic experiments [27]. Sequential positive and negative selection may reduce hematopoietic, endothelial, dead-cell, and debris contamination before more precise analysis. Functionalized magnetic particles, multivalent capture interfaces, and cleavable binding systems may further improve capture or facilitate gentler release. Reporter-based immunomagnetic separation targeting SOX2/OCT4-responsive cells illustrates how magnetic enrichment can be extended beyond conventional surface markers [28]. However, MACS generally resolves fewer simultaneous features than FACS, and bead-associated recovery does not establish self-renewal or tumor-initiation capacity. It should therefore be regarded primarily as a pre-enrichment or depletion step rather than as definitive evidence of BCSC identity.

A rational conventional workflow is thus MACS pre-enrichment followed by multiparameter FACS and independent functional confirmation. Post-sort evaluation should distinguish purity, viability, and stemness because these outcomes are related but not interchangeable. Mammosphere culture was originally developed to propagate mammary stem and progenitor cells under non-adherent conditions and remains widely used as an initial self-renewal assay [29]. However, mammosphere-forming efficiency and apparent self-renewal capacity vary across primary breast tumors, normal breast tissues, and established cell lines [30]. The assay is also sensitive to aggregation, plating density, proliferation rate, and culture adaptation and should therefore not be treated as definitive evidence of stemness. Serial sphere formation, differentiation assays, and clonogenic measurements provide stronger evidence than a single sphere-forming endpoint, while limiting-dilution xenotransplantation remains the more stringent test of tumor-initiating capacity [31]. Microfluidic mammosphere systems can improve control of clonality and enable recovery of functionally selected tumor-initiating populations for downstream validation [32]. Accordingly, the value of conventional sorting lies not in a single purity estimate, but in an integrated process that connects subtype-adapted phenotypic enrichment with cell-state preservation and functional verification. Only viable and functionally confirmed BCSCs provide a defensible donor-cell source for subsequent collection and interpretation of BCSC-derived EVs.

These functional assays should be interpreted as a hierarchy rather than as interchangeable endpoints. Mammosphere formation is suitable for initial screening but is sensitive to aggregation, plating density and proliferation; patient-derived organoids provide an intermediate, clinically contextualized tier for clonal growth and drug-response assessment; and limiting-dilution xenotransplantation remains the most stringent in vivo test of tumor-initiating capacity, albeit with low throughput, long duration and high cost. Phenotypic enrichment should therefore be paired with at least one independent functional assay, with the validation tier matched to the strength of the biological claim. Microfluidic organ-on-chip systems combined with breast-cancer patient-derived organoids further illustrate how functional testing can be standardized in limited specimens [33].

### 2.2. Microfluidic Sorting Technologies

Microfluidic platforms offer particular value for BCSC research because they can process limited specimens in enclosed channels while integrating cell focusing, separation, imaging, molecular detection, and recovery. These features may reduce transfer loss and reagent consumption when handling core biopsies, small metastatic specimens, or rare circulating and disseminated tumor cells. However, miniaturization does not itself provide BCSC specificity. A platform should be considered BCSC-compatible only when it preserves viable cells, allows downstream recovery, and connects the selected physical or molecular phenotype with independent stemness validation. Results obtained from cultured cells spiked into buffer or healthy blood therefore cannot be directly extrapolated to dissociated clinical tumors containing stromal cells, debris, apoptotic cells, and heterogeneous tumor-cell clusters.

#### 2.2.1. Passive Microfluidic Sorting

Passive microfluidic sorting exploits intrinsic biophysical differences among cells and therefore enables label-free enrichment without antibodies, magnetic particles, or external force fields. Inertial focusing and Dean-flow devices use lift and drag forces, whereas deterministic lateral displacement arrays and microfilters route or retain cells according to effective size and mechanical passage through defined gaps. A microbarrier platform enriched highly deformable SUM149 breast cancer cells that displayed increased mammosphere formation and other stem-like characteristics [34]. Hydrodynamic shear has also been used to isolate weakly adherent breast cancer fractions with enhanced ALDH activity and tumorigenic potential [35]. More recently, a tandem mechanical system combined sequential selection for high deformability and low substrate adhesion to enrich cancer stem-like cells and support drug screening [36]. These studies demonstrate that mechanical phenotypes can enrich BCSC-like populations, but they do not establish deformability or adhesion as universal BCSC markers. EMT, treatment exposure, cell-cycle state and tissue processing can alter the same physical properties; accordingly, passive devices should be reported as pre-enrichment tools and validated against subtype-adapted molecular markers and stemness assays rather than presented as stand-alone BCSC identifiers.

#### 2.2.2. Active Microfluidic Sorting

Active microfluidic sorting applies externally controlled acoustic, electrical, magnetic, or optical forces to manipulate cells with greater spatial and temporal precision than passive systems. Acoustic microfluidics, for example, uses acoustic radiation forces and streaming to move cells without permanent labeling or direct mechanical contact. Recent acoustofluidic systems have enriched cancer cells directly from whole blood through acoustic-field-induced cell organization [37]. A two-step acoustic workflow based on cell size and acoustic impedance has further generated viable circulating tumor cell (CTC)-enriched fractions suitable for downstream single-cell analysis [38]. Because acoustic response depends on cell size, density, compressibility, medium composition, flow rate, and acoustic frequency, separation conditions must be optimized for each sample type. Importantly, most acoustic studies have focused on general tumor-cell or CTC enrichment rather than functionally verified BCSCs. Acoustic platforms should therefore report post-sort viability, recovery, thermal exposure, cluster integrity, and stemness rather than separation efficiency alone.

Dielectrophoresis separates cells according to their frequency-dependent polarization in a non-uniform electric field. Differences in membrane capacitance, cytoplasmic conductivity, cell size, and medium conductivity can support label-free enrichment. A three-dimensional-electrode DEP system has separated MDA-MB-231 breast cancer cells from mixed cell populations while preserving cell viability [39]. Integrated DEP platforms can also capture individual breast cancer cells for downstream single-cell biochemical analysis [40]. Other active approaches combine microfluidics with magnetic particles, optical forces, impedance sensing, or multimodal imaging. Immunomagnetic microfluidics has also been used to isolate HER2-positive breast cancer cells through antibody-functionalized magnetic particles [41]. Nevertheless, electrical and magnetic platforms are not intrinsically BCSC-specific, and their outputs require correlation with multiparameter markers and functional assays.

When used sequentially, passive microfluidic modules are best positioned as high-throughput, label-free first-stage pre-enrichment, whereas active modules provide a more controllable second-stage enrichment at the cost of greater hardware and parameter complexity. Neither stage independently establishes BCSC identity; both should feed into multiparameter phenotyping and functional confirmation.

#### 2.2.3. Performance Evaluation and Integrated Workflows

Cross-study comparison should be organized around five fixed engineering dimensions: purity, recovery yield, viability, throughput and stemness preservation. Purity is the proportion of target-defined cells among recovered cells, whereas recovery yield is the proportion of input target cells successfully recovered; these denominators must not be interchanged. Viability, throughput, processing time, clogging, cluster preservation, cell-release efficiency, sterility, and operator dependence should be reported separately. Stemness maintenance cannot be inferred from recovery or marker expression and should be examined after sorting using serial mammosphere formation, differentiation, or tumor-initiation assays. Microfluidic mammosphere platforms illustrate how cell isolation can be connected directly with clonality and functional assessment [32]. Comparisons remain unreliable when one study reports recovery from spiked cell lines and another reports marker-defined purity from patient tissue.

The most promising near-term role of microfluidics is therefore integrated pre-enrichment and sample preparation rather than replacement of FACS-based BCSC definition. Label-free modules can reduce abundant non-target cells, affinity or magnetic regions can improve molecular specificity, and on-chip imaging can document individual cells before recovery. This strategy is supported by microfluidic filtration studies that recovered rare disseminated tumor cells from the bone marrow of patients with breast cancer [42]. Subsequent patient-based work confirmed that viable disseminated cells could be enriched for downstream molecular characterization [43]. However, DTC or CTC recovery does not demonstrate that the isolated cells are BCSCs. A clinically translatable workflow should therefore combine microfluidic pre-enrichment with multiparameter phenotyping and post-sort functional validation. Microfluidics can improve sample utilization, automation, and cell recovery, but overlapping physical properties and limited direct BCSC validation mean that it should complement rather than replace molecular and functional criteria.

### 2.3. Affinity Capture and Single-Cell Sorting Platforms

#### 2.3.1. Affinity-Based Capture Platforms

Affinity-based platforms improve the molecular specificity of cell enrichment by immobilizing antibodies, peptides, or other ligands on microbeads, microposts, channel walls, hydrogels, or nanostructured substrates. Antibody-coated micropost devices established that viable rare tumor cells can be captured directly from unprocessed blood under controlled microfluidic flow [44]. Multiparameter interfaces can further reduce dependence on a single epithelial marker and accommodate phenotypic heterogeneity [45]. For BCSC research, however, capture through EpCAM, CD44, CD49f, or HER2 represents phenotypic enrichment rather than definitive identification. Antibody affinity, epitope masking, receptor internalization, clone variability, and difficult cell release may also affect recovery and downstream function. Affinity devices should therefore incorporate negative-selection markers, viability assessment, gentle release, and post-capture stemness validation.

Aptamers provide an alternative recognition element because they are chemically synthesized, relatively small, and amenable to sequence-defined modification and reversible release. The MS03 aptamer was selected against mammosphere-derived breast cancer cells and proposed as a marker for a stem-like breast cancer population [46]. EpCAM-binding DNA aptamers have also been developed for the recognition of epithelial tumor and stem-cell-associated phenotypes [47]. Multivalent aptamer cocktails targeting epithelial and mesenchymal markers can improve the capture of heterogeneous breast cancer cells or CTCs [48]. Nevertheless, aptamers selected against cultured cell lines may bind culture-dependent epitopes, and nuclease degradation or nonspecific electrostatic interactions may reduce performance in clinical specimens. Aptamers should therefore be considered tunable affinity reagents rather than intrinsically BCSC-specific probes.

Nanostructured interfaces increase ligand–cell contact by providing high surface area, local topographical confinement, and multivalent interactions. Nanostructure-based chips have supported breast cancer cell capture followed by on-device drug-response assessment [49]. A graphene-oxide-interfaced microfluidic system has enabled antibody-conjugated capture and multiplex detection of circulating tumor cells and circulating tumor microemboli [50]. However, the material interface is not biologically inert. Surface stiffness, roughness, ligand spacing, and chemical composition can alter integrin signaling, cytoskeletal tension, proliferation, EMT, and stemness. Matrix stiffness has been shown to influence BCSC-associated phenotypes and treatment sensitivity [51]. Consequently, ligand-free controls, flat-surface controls, limited residence time, and post-release phenotyping are necessary to distinguish pre-existing BCSCs from states induced by the capture substrate.

Recognition-element selection should be treated as an engineering trade-off. Antibodies offer mature high-affinity and high-specificity reagents for known subtype markers, but their large size, production cost, lot variability, epitope masking and difficult release can reduce recovery. Aptamers are sequence-defined, comparatively inexpensive to synthesize, readily multiplexed and compatible with reversible binding, but nuclease degradation, folding dependence and nonspecific electrostatic interactions require control in blood and tissue digests. Peptides are compact, chemically stable and relatively low-cost, which favors dense surface display, although their affinity and BCSC selectivity are often lower or more model-dependent than those of validated antibodies [52]. Reversible interfaces—including pH-, enzyme-, light- or temperature-responsive linkers and dynamically assembled affinity layers—can decouple efficient capture from gentle cell release, thereby improving viability and downstream culture or molecular analysis [53]. For needle-biopsy material with a defined subtype and very low cell input, validated antibody or nuclease-protected aptamer panels provide the strongest molecular specificity; for heterogeneous CTC or other circulating rare-cell populations, multiplex aptamer or peptide interfaces may reduce steric crowding and reagent cost but require stricter off-target and matrix testing. All three approaches should report absolute recovered-cell number, background-cell depletion, release efficiency, viability and compatibility with functional validation rather than capture efficiency alone.

#### 2.3.2. Single-Cell Sorting and Functional Analysis

Droplet, microwell, and single-cell microfluidic systems extend cell sorting from marker enrichment to functional analysis. Single-cell co-culture chips can control clonality and monitor sphere formation from individual breast cancer cells [54]. Three-dimensional gel-island platforms have similarly enabled isolation and longitudinal tracking of single stem-like breast cancer cells [55]. Functional microfluidic selection of migratory breast cancer cells identified populations with enhanced tumor-initiation and metastatic capacity in vivo [32]. Recoverable cells can subsequently undergo scRNA sequencing, protein analysis, chromatin profiling, lineage tracing, or drug testing. Single-cell transcriptomics of microfluidically enriched migratory breast cancer cells has demonstrated that phenotypically similar cells can retain substantial molecular heterogeneity [56]. Nevertheless, transcriptomic clustering or expression of a stemness program cannot substitute for serial self-renewal or tumor-initiation assays.

The most informative platforms will therefore integrate affinity recognition with single-cell recovery and functional readouts. Linking the phenotype, clonal behavior, treatment response, and EV secretion of the same cell would make it possible to determine whether a rare BCSC state disproportionately contributes to vesicle-mediated resistance or microenvironmental remodeling. A droplet-array platform has recently demonstrated single-cell EV analysis in HER2-positive breast cancer cells during trastuzumab exposure [57]. Although such systems do not yet establish BCSC-specific EV secretion, they provide an engineering basis for pairing verified BCSC and non-BCSC clones with their vesicular outputs. The core performance of all BCSC isolation platforms discussed in Section 2.1, Section 2.2 and Section 2.3, including separation basis, application scenario, advantages and limitations, is compared in Table 1. Thus, affinity capture improves molecular enrichment, whereas single-cell platforms establish heterogeneity and functional traceability; their integration is a key step toward source-defined BCSC-EV research.

### 2.4. Minimum Validation Standards for BCSC-Derived EV Attribution

To standardize the attribution of BCSC-derived EVs, we propose a three-tier validation framework based on the strength of donor-cell evidence. Tier 1 (BCSC-associated EVs) refers to EVs obtained from cell populations enriched using single or dual BCSC markers, such as CD44^high^/CD24^low^ or ALDH-high, without independent functional validation of stemness. Tier 2 (minimum standard for BCSC-derived EV attribution) requires multiparametric phenotypic characterization together with at least one independent in vitro functional assay of stemness, such as serial mammosphere formation or patient-derived organoid self-renewal. Tier 3 (high-confidence BCSC-derived EVs) requires orthogonal phenotypic characterization combined with limiting-dilution xenotransplantation and/or lineage tracing, together with assessment of donor-cell purity and viability. Under this framework, the term “BCSC-derived EVs” should be reserved for studies meeting Tier 2 or Tier 3 criteria, whereas EVs characterized only by Tier 1 evidence should be referred to as “BCSC-associated EVs”.

## 3. Engineering-Based Characterization and Functional Research of BCSC-Associated Extracellular Vesicles

**Figure 2:**
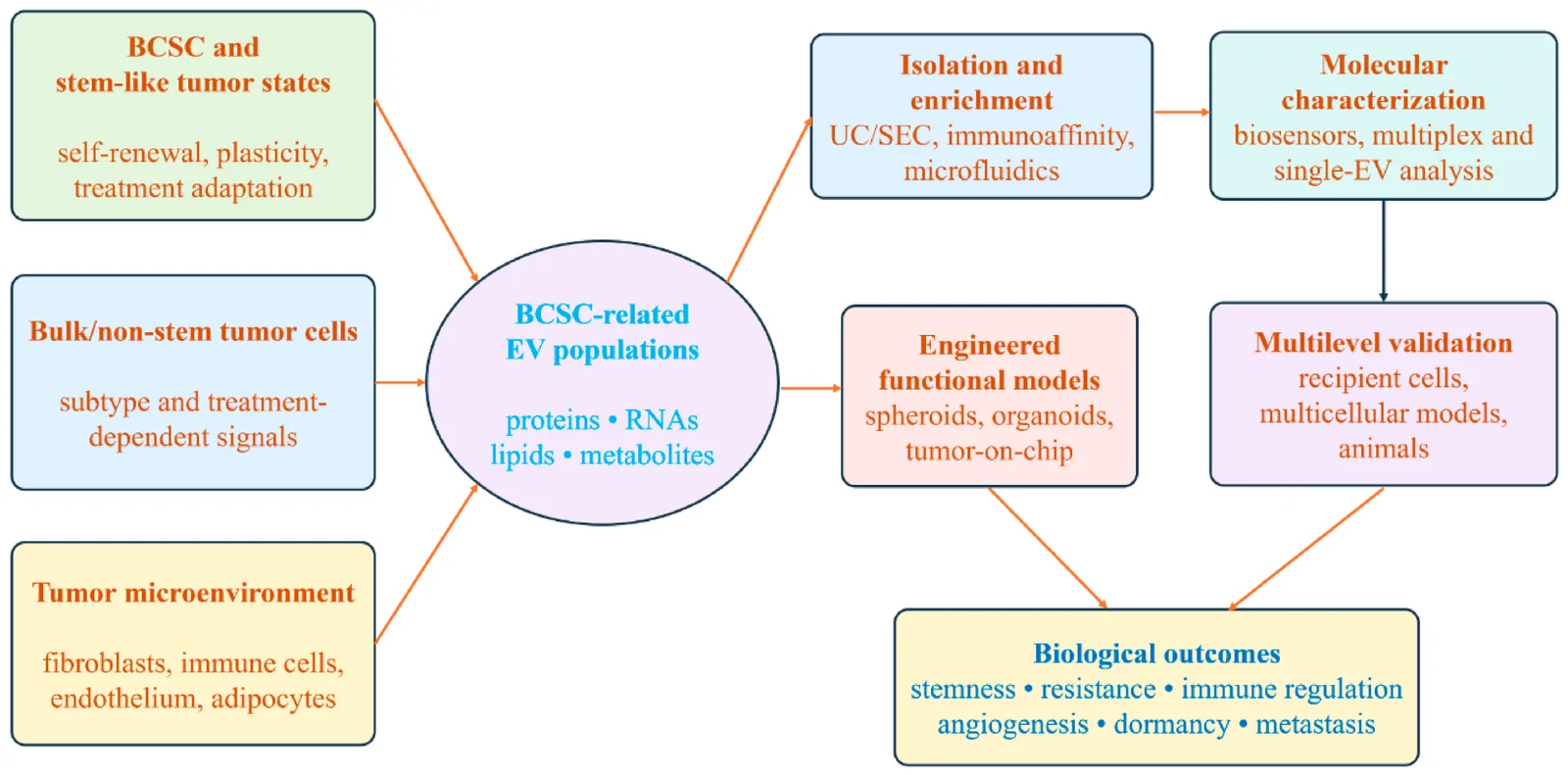
Engineering framework for characterization and functional analysis of BCSC-related extracellular vesicles. BCSC-related extracellular vesicle populations may originate from functionally verified BCSCs and stem-like tumor states, bulk or non-stem tumor cells, and components of the tumor microenvironment. These vesicles carry proteins, RNAs, lipids, and metabolites that reflect donor-cell identity, breast cancer subtype, treatment exposure, and microenvironmental conditions. Isolation and enrichment are followed by molecular characterization using biosensors, multiplex assays, and single-vesicle technologies. Engineered functional models, including spheroids, organoids, and tumor-on-chip systems, together with recipient-cell, multicellular, and animal validation, are used to assess biological effects on stemness, treatment resistance, immune regulation, angiogenesis, dormancy, and metastasis. The framework emphasizes bidirectional vesicle-mediated communication between tumor cells and the surrounding microenvironment. BCSC, breast cancer stem cell; EV, extracellular vesicle; UC, ultracentrifugation; SEC, size-exclusion chromatography.

Accurate interpretation of BCSC-associated EV biology depends on a source-resolved analytical workflow encompassing vesicle isolation, molecular characterization, and hierarchical functional validation. The corresponding experimental framework is outlined in Figure 2.

### 3.1. Biogenesis and Distinctive Features of BCSC-Associated Extracellular Vesicles

Extracellular vesicles generated through the endosomal pathway are commonly termed exosomes; however, this designation should be used only when endosomal origin is experimentally supported. Following inward budding of the endosomal membrane, intraluminal vesicles accumulate within multivesicular bodies, which subsequently fuse with the plasma membrane and release their vesicular contents into the extracellular space [58]. This process is coordinated by both endosomal sorting complex required for transport (ESCRT)-dependent and ESCRT-independent mechanisms involving tetraspanins, ceramide, membrane lipid microdomains, Rab GTPases, and other trafficking-associated proteins [59]. In breast cancer stem cells (BCSCs), EV biogenesis and cargo loading are further influenced by the intrinsic stem-like state of the donor cells and by microenvironmental conditions such as hypoxia, oxidative stress, acidic pH, nutrient deprivation, and therapeutic exposure.

Compared with the bulk breast cancer cell population, EVs released from BCSC-enriched populations have been reported to contain distinct combinations of nucleic acids, proteins, lipids, and metabolic components associated with self-renewal, survival, epithelial–mesenchymal plasticity, and resistance to cellular stress [60,61,62]. Their distinctive features are unlikely to be defined by a single universal molecule. Instead, they appear to reflect the coordinated enrichment of cargo associated with stemness-related signaling and adaptive programs operating within the donor cells. Breast cancer subtype, genetic background, treatment history, and local microenvironment may further shape EV composition, contributing to substantial heterogeneity among BCSC-associated EV populations.

Functionally, BCSC-associated EVs can act through both autocrine and paracrine signaling. Within stem-like tumor cell populations, they may reinforce self-renewal, survival, and phenotypic plasticity. Following uptake by more differentiated breast cancer cells, EV cargo may activate stemness-associated pathways and promote the acquisition of BCSC-like characteristics. These vesicles have also been implicated in epithelial–mesenchymal transition, invasion, metastatic dissemination, and therapeutic resistance. Notably, extracellular vesicles released by chemotherapy-exposed breast cancer cells can promote stem-like and treatment-resistant phenotypes in neighboring cells [10], suggesting that vesicle-mediated communication contributes to the dynamic expansion or redistribution of stem-like tumor cell populations during treatment.

**Figure 3:**
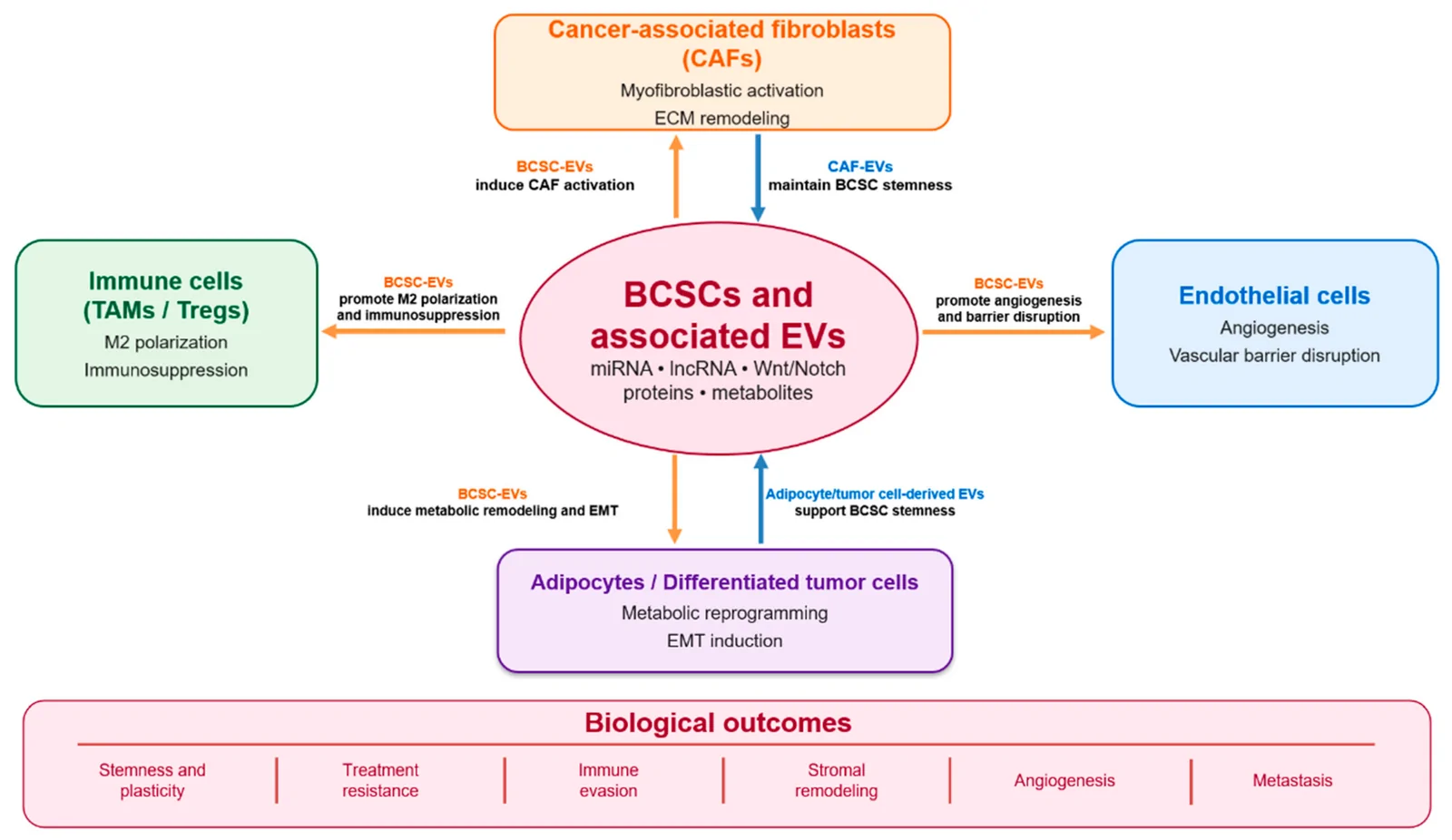
Bidirectional communication between BCSCs and the tumor microenvironment mediated by extracellular vesicles. BCSC-associated EVs influence cancer-associated fibroblasts (CAFs), immune cells, endothelial cells, adipocytes, and differentiated tumor cells, promoting stromal remodeling, immune suppression, angiogenesis, metabolic reprogramming, and EMT. Reciprocal signaling from the tumor microenvironment can support BCSC stemness and plasticity. These interactions contribute to treatment resistance, immune evasion, and metastasis.

BCSC-related EV communication also extends to the tumor microenvironment (TME). By influencing stromal cells, immune cells, endothelial cells, and other niche components, they may support angiogenesis, immune evasion, metabolic adaptation, tumor dormancy, and metastatic niche formation. BCSC-associated EVs can promote myofibroblastic cancer-associated fibroblast (CAF) activation, M2 polarization and immunosuppression, angiogenesis and vascular barrier disruption, and metabolic reprogramming and EMT in adipocytes or differentiated tumor cells. CAF-derived EVs may in turn support BCSC stemness. Mesenchymal stromal cell-derived vesicles have been associated with metastatic dormancy [63], whereas adipocyte-derived vesicles can enhance aggressive breast cancer behavior [9]. These observations also indicate that EV-mediated communication is bidirectional: BCSCs can modify their surrounding niche through secreted vesicles, while microenvironment-derived vesicles can induce or stabilize stem-like states in breast cancer cells. These reciprocal interactions connect BCSCs with stromal, immune, vascular, and tumor-cell compartments of the TME (Figure 3).

Collectively, BCSC-associated EVs represent dynamic extensions of the molecular and functional state of BCSCs rather than a uniform vesicle population. Their composition and biological activity are jointly shaped by intrinsic stemness programs, breast cancer heterogeneity, and environmental stress. This complexity underscores the importance of engineering-based platforms capable of selectively isolating, detecting, and characterizing BCSC-associated EV subpopulations.

A source-attribution caveat remains important because donor-cell impurity can obscure EV molecular profiles, generate conflicting functional conclusions, and reduce cross-study comparability. Accordingly, attribution of BCSC origin should follow the validation criteria defined in Section 2.4, together with standardized culture conditions and orthogonal EV characterization [15].

### 3.2. Engineering-Based Isolation and Detection of BCSC-Related EVs

Before comparing EV isolation and detection platforms, it is important to recognize that their performance is strongly dependent on the sample matrix. As emphasized in the MISEV2023 guidelines, pre-analytical variables related to donor-cell condition, sample collection, processing, and storage can substantially influence EV abundance, composition, and downstream measurements [15]. EVs collected from conditioned medium are affected by donor-cell state, cell density, viability, medium composition, and exposure to hypoxia or treatment. Such cell-line and other controlled experimental systems are useful for establishing analytical feasibility, but they do not reproduce the cellular and vesicular complexity of patient-derived tissues or clinical blood. Evidence from PDX models or primary tissues provides a closer representation of tumor heterogeneity, although such samples remain less extensively evaluated for BCSC-associated EV isolation. Clinical plasma, serum, and whole blood present the greatest analytical complexity because tumor-associated EVs coexist with abundant vesicles from platelets and other host cells and are additionally influenced by sample handling and storage [64,65]. These differences can alter EV recovery and analytical background, thereby complicating the detection of low-abundance BCSC-related signals. Accordingly, isolation or detection performance established using cell lines or spiked samples cannot be assumed to represent performance in untreated clinical blood. The choice of separation and detection strategy should be adapted to the biological source of the sample and the intended downstream analysis.

#### 3.2.1. Conventional EV Separation Methods

Conventional methods provide different balances between recovery, purity, and functional preservation. Differential ultracentrifugation is widely used but may co-pellet proteins and lipoproteins; density gradients improve specificity but are laborious. Size-exclusion chromatography is gentle and reproducible, although similarly sized particles may remain. Ultrafiltration concentrates EVs but introduces membrane-dependent loss, whereas polymer precipitation produces high apparent recovery with substantial contamination. Immunoaffinity capture enriches a marker-defined subset but cannot represent the complete EV population. A recent direct comparison found that ultrafiltration followed by size-exclusion chromatography provided higher yield and reproducibility than sucrose-cushion ultracentrifugation, although performance remained dependent on sample type and downstream endpoint [66]. Evidence from clinical biofluids further demonstrates the importance of sample matrix: studies using human serum and plasma have shown that the selected isolation workflow changes EV protein, RNA, and functional readouts [67]. These clinical-biofluid results should not be directly compared with recovery or purity estimates obtained from conditioned medium or spiked samples unless the sample matrix and analytical denominators are comparable. Method choice should therefore follow the downstream question rather than a universal hierarchy.

#### 3.2.2. Microfluidic Isolation and Integrated Processing

Microfluidic platforms integrate separation, concentration, and detection while reducing sample volume and transfer loss. Size-based devices use membranes, nanostructures, or hydrodynamics, whereas affinity devices use antibodies or aptamers; acoustic and electrokinetic modules provide label-free manipulation.

Initial feasibility has largely been established in controlled experimental systems. The μCENSE platform demonstrated rapid centrifugal extraction of EVs [68]. A nanostructured SiO2 chip captured CD63- and CD81-positive EVs from breast-cancer conditioned medium [69]. These studies establish technical feasibility under controlled conditions but do not demonstrate equivalent performance in patient blood. Evidence from PDX or primary breast-tumor specimens remains comparatively limited for these integrated EV-isolation platforms. This gap is important because tumor-derived material introduces cellular heterogeneity and biological contaminants that are not reproduced by cell-line conditioned medium or simple spiking experiments. Clinical-biofluid studies have extended microfluidic EV isolation to substantially more complex matrices. The cleavable-linker HBEXO-Chip enabled capture and release of EpCAM-positive breast-cancer-associated EVs from blood [70]. The Sub-ExoProfile chip separated CD81-, EpCAM-, and HER2-defined EV subpopulations for proteomic analysis [71]. These systems improve access to rare tumor-associated vesicles, but EpCAM or HER2 positivity does not establish BCSC origin. A dielectrophoresis-based chip enabled rapid capture and dual-marker analysis of small EVs from 20 to 50 μL clinical plasma [72]. Cascaded microfluidic filtration has isolated total EVs directly from whole blood within 30 min [73]. Clinical translation will require reproducible performance across sample volumes, batches, and independent cohorts. Importantly, successful isolation of total EVs from whole blood or marker-defined breast-cancer-associated EVs from plasma does not establish recovery of BCSC-derived EVs. No platform has yet demonstrated direct recovery of EVs from functionally verified BCSCs in untreated whole blood; this remains a specific source-resolution challenge.

#### 3.2.3. Biosensing and Single-EV Analysis

Biosensors translate EV binding or cargo into optical, plasmonic, electrochemical, fluorescent, or Raman signals. Analytical development has included controlled EV preparations, but the principal translational evidence in breast cancer comes from plasma and serum studies. Evidence using PDX-derived or primary-tissue-derived EVs as an intermediate validation step remains limited. At the clinical-biofluid level, fiber-optic surface plasmon resonance has detected breast-cancer-associated EVs directly in plasma [74]. Electrochemical sensors offer compact instrumentation but remain vulnerable to fouling and matrix interference. Raman and surface-enhanced Raman spectroscopy provide label-free molecular fingerprints. An artificial-intelligence-assisted SERS platform distinguished serum EV profiles from breast cancer and control samples [75]. Raman analysis has also been explored for monitoring treatment-related EV changes in HER2-positive breast cancer [76]. However, spectral models require locked preprocessing, blinded testing, and external validation because batch effects and data leakage may inflate performance. At present, most biosensing platforms characterize broader breast-cancer-associated EV patterns, and their capacity to resolve BCSC-associated subpopulations remains to be established.

Single-EV analysis addresses heterogeneity hidden by bulk measurements and can determine whether tumor, stemness, and functional markers coexist on the same vesicle. High-resolution flow cytometry, imaging, digital immunoassays, and Raman methods can profile rare EV subsets. A community compendium has defined controls for swarm detection, fluorescence sensitivity, calibration, and reporting [77]. Cross-platform comparability can be improved by converting fluorescence and light-scatter signals into standardized units through instrument calibration [78]. The ddSEE system concurrently detected an EV surface protein and an internal miRNA at single-vesicle resolution [79]. Multiparametric imaging has also demonstrated molecular heterogeneity within HER2-enriched EV populations [14]. Multiparameter co-detection of breast-cancer-associated surface features, stemness-related markers, and functional cargos may improve the resolution of BCSC-related EV subpopulations. Clinical application of this approach has begun to emerge: automated dual-image analysis has enabled quantitative single-vesicle profiling of EpCAM- and CD24-positive plasma EV fractions across HER2-positive breast-cancer stages [80]. However, co-detection of these markers identifies an associated phenotype and does not independently establish BCSC origin.

Together, conventional separation methods, microfluidic platforms, biosensors, and single-EV technologies provide complementary approaches for the isolation and molecular characterization of BCSC-related EVs. The technical principles, applicable scenarios, advantages and limitations of these EV isolation and detection platforms are detailed and compared in Table 2. Their integration can improve sample utilization, analytical sensitivity, and the resolution of rare vesicle subpopulations. Performance should nevertheless be interpreted according to the biological source and sample matrix in which it was established. However, molecular detection alone cannot determine how these EVs influence tumor cells or the surrounding microenvironment. Addressing this question requires engineered functional models that reproduce the spatial, temporal, and multicellular features of EV-mediated communication.

### 3.3. Functional Analysis of BCSC-Associated EVs in Engineered Models

#### 3.3.1. Cell-Line and Controlled Experimental Models

Conventional two-dimensional cultures provide controlled conditions for studying EV uptake and intracellular signaling but poorly reproduce the extracellular matrix, cell polarity, oxygen and nutrient gradients, fluid transport, and multicellular interactions of breast tumors. They also commonly expose recipient cells to a single high EV dose, whereas intercellular vesicle communication in vivo is continuous and spatially restricted. Three-dimensional mammospheres and tumor spheroids partly address these limitations by generating cell–cell contacts, diffusion gradients, and stem-like phenotypes. However, spheroids may serve either as BCSC-enriched donor models or as recipient models, and these roles should be distinguished. Sphere culture itself can modify cellular stress, metabolism, and EV release; therefore, EVs obtained from mammospheres should not automatically be considered verified BCSC-derived EVs.

More complex engineered models can add controlled perfusion, extracellular-matrix architecture, oxygen and nutrient gradients, and defined multicellular compartments to cell-line experiments. A three-dimensional human liver-chip showed that breast-cancer-derived EVs activated liver sinusoidal endothelial cells, disrupted vascular-barrier properties, and increased subsequent tumor-cell adhesion through EV-associated TGF-β1 signaling [82]. Such models can therefore connect vesicle exposure with organ-specific metastatic events under physiologically relevant flow. However, most current chips use breast cancer cell-line EVs rather than vesicles from functionally verified BCSCs. A perfusable hydrogel vessel-like breast-cancer-on-chip model provides an additional engineering basis for studying transport and tumor-cell propagation under flow [83].

Engineered three-dimensional systems are particularly useful for resolving how distinct tumor-cell states remodel the microenvironment. In a TNBC model, EVs isolated from cancer stem-like and differentiated cancer-cell populations carried different cargos and produced different stromal responses [84]. EVs from differentiated tumor cells activated secretory CAF programs that supported maintenance of the CSC phenotype, whereas EVs from the stem-like fraction promoted myofibroblastic CAF activation, endothelial remodeling, and formation of a lung environment permissive for metastatic growth. This study illustrates that BCSC-related EV communication is not unidirectional (Figure 3): non-stem tumor-cell EVs may sustain BCSCs through stromal intermediates, while EVs from the stem-like fraction may prepare vascular and metastatic niches. Together with evidence for EV-mediated interactions involving immune and adipose-lineage cells, these findings support a reciprocal BCSC–TME signaling network in which EVs can both transmit stem-like tumor-cell signals to the surrounding niche and contribute to feedback that maintains or induces stem-like tumor states. Co-culture models should therefore include tumor cells, fibroblasts, endothelial cells, and, where feasible, immune or adipose-lineage cells.

Drug-resistance studies should distinguish EV-mediated changes in general survival from effects specifically involving stemness. EVs from tamoxifen-resistant breast cancer cells increased resistance, altered stemness-marker expression, and enhanced sphere formation in recipient cells through transfer of miR-9-5p [85]. Earlier work also demonstrated transfer of P-glycoprotein-associated drug resistance from resistant to sensitive breast cancer cells through vesicles [86]. Such models should report the donor-cell state, treatment conditions, EV dose, recipient-cell viability, apoptosis, drug IC50, and functional stemness endpoints. Likewise, migration or invasion in a two-dimensional assay should not be described as metastasis. Stronger metastatic evidence requires sequential evaluation of invasion, endothelial crossing, distant-organ adhesion, colonization, and in vivo metastatic growth. Recent breast-cancer studies have further shown that EVs from drug-resistant cells can transfer resistance-associated transcriptional programs and remodel recipient-cell or macrophage responses [87,88]. Collectively, these cell-line and engineered-model studies establish biological plausibility and provide systems for mechanistic perturbation, but they do not by themselves establish that the same EV-mediated effects occur in patient-derived tumors or clinical biofluids.

#### 3.3.2. PDX and Primary-Tissue Models

Patient-derived organoids provide a more clinically relevant platform because they retain portions of the histological, genetic, and receptor heterogeneity of the tumors from which they originate [89]. Breast cancer organoids have also reproduced interpatient differences in therapeutic response and have been investigated as predictors of neoadjuvant chemotherapy sensitivity [90]. In BCSC-EV studies, organoids can be used to determine whether vesicles alter clonal growth, lineage composition, treatment response, and the expansion of stem-like populations within a patient-specific background. Organoid microarrays can further increase experimental throughput and reduce variability in three-dimensional drug testing [91]. Nevertheless, organoid establishment may select particular clones, and commonly used matrices introduce undefined growth factors. Most epithelial organoids also lack complete vascular, stromal, and immune compartments, limiting their ability to reproduce systemic EV effects. Recent Bioengineering work combining patient-derived breast-cancer organoids with a microfluidic organ-on-chip platform demonstrates how limited clinical specimens can support more standardized functional drug evaluation [33]. A second Bioengineering study showed that three-dimensional growth geometry and mechanical stress can alter breast-cancer stem-cell and EMT-marker expression, emphasizing that model architecture itself must be controlled when interpreting EV-induced phenotypes [92]. Patient-derived systems therefore provide an important intermediate level between controlled cell-line experiments and clinical biofluid studies. However, direct functional studies using EVs from rigorously validated BCSCs isolated from primary breast tumors or PDX models remain limited. In particular, the ability of a patient-derived model to preserve tumor heterogeneity does not verify the BCSC origin of the EV preparation.

#### 3.3.3. Clinical Biofluids

The final level of evidence involves EVs obtained directly from clinical biofluids. Compared with conditioned medium or engineered culture systems, plasma and serum contain heterogeneous EV populations from tumor and non-tumor sources, making both source attribution and causal functional analysis substantially more difficult. Current clinical studies predominantly characterize breast-cancer-associated EV profiles or marker-defined EV subpopulations and typically do not establish direct origin from functionally verified BCSCs. Consequently, the mechanistic effects demonstrated in cell-line, spheroid, organoid, or chip models should not be assumed to represent the biological activity of a purified BCSC-derived EV population in patient blood. Functional validation using source-resolved clinical EV populations remains an important gap between experimental BCSC-EV biology and clinical translation.

#### 3.3.4. Causal Validation Across Evidence Levels

Causal attribution requires more than observing that EV exposure changes a recipient phenotype. Appropriate experiments include EV-depleted conditioned medium, mock isolation, inhibition or genetic disruption of EV release, blockade of vesicle uptake, depletion of the proposed cargo, inhibition of the downstream pathway, and rescue with the restored cargo or purified vesicles. Orthogonal EV isolation and characterization are needed to exclude soluble mediators and non-vesicular particles [15]. Functional conclusions should then be tested across increasing levels of complexity, from two-dimensional cells to spheroids, organoids, perfused chips, and animal models. Where clinical biofluid samples are used, source attribution and matrix-specific controls are additionally required before experimental mechanisms can be extended to circulating BCSC-associated EV signals. Engineered models therefore improve physiological relevance and temporal control, but only donor-cell verification, perturbation experiments, and multilevel validation can establish a defensible causal chain between a functionally verified BCSC donor state, its EVs and stemness, resistance, or metastatic progression.

Source-resolved functional studies in organoids and tumor-on-chip systems can identify candidate vesicular cargos, surface features, and uptake mechanisms with translational relevance. These findings provide two complementary starting points: the development of BCSC-associated EV signatures for treatment-response and recurrence monitoring; the engineering of EV-based or biomimetic systems to target resistant tumor states. Representative biological and translational studies of BCSC-associated and breast-cancer-related EVs are summarized in Table 3.

## 4. Translational Applications of Engineered Extracellular Vesicles Targeting BCSCS

**Figure 4:**
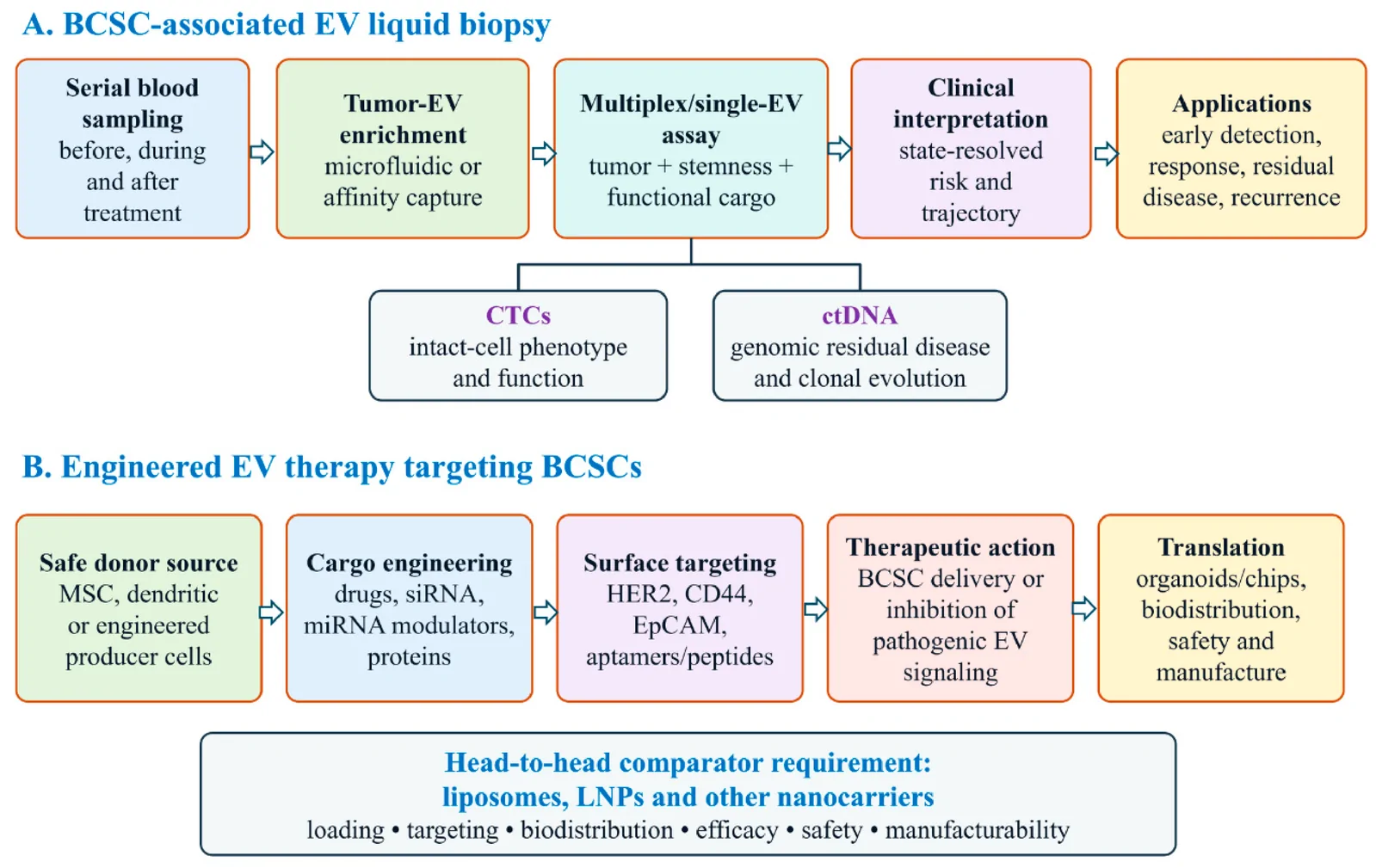
Translational pathways for BCSC-associated extracellular vesicle diagnostics and BCSC-targeted therapy. (**A**) In the diagnostic pathway, serial blood sampling is followed by enrichment of tumor-associated extracellular vesicles and multiplex or single-vesicle assessment of tumor-associated, stemness-related, and functional cargo. Integrated interpretation may support longitudinal evaluation of treatment response, residual disease, recurrence risk, and disease trajectory. BCSC-associated extracellular vesicles are intended to complement rather than replace circulating tumor cells and circulating tumor DNA, which provide intact-cell phenotypic information and tumor-genomic information, respectively. (**B**) In the therapeutic pathway, extracellular vesicles from qualified donor cells may be engineered with therapeutic cargos and surface-targeting ligands to deliver drugs, nucleic acids, or proteins to resistant stem-like tumor states or to disrupt pathogenic vesicle signaling. Translation requires validation in organoid and microphysiological models, biodistribution studies, safety assessment, and scalable manufacturing. Direct comparisons with liposomes, lipid nanoparticles, and other nanocarriers are required to determine relative advantages in cargo loading, targeting, biodistribution, efficacy, safety, and manufacturability. BCSC, breast cancer stem cell; EV, extracellular vesicle; CTC, circulating tumor cell; ctDNA, circulating tumor DNA; MSC, mesenchymal stromal cell; siRNA, small interfering RNA; miRNA, microRNA; HER2, human epidermal growth factor receptor 2; EpCAM, epithelial cell adhesion molecule; LNP, lipid nanoparticle.

Building on these cell-sorting and vesicle-characterization platforms, BCSC-associated EVs show translational potential in two complementary directions: noninvasive liquid biopsy and targeted therapeutic intervention. The corresponding pathways are summarized in Figure 4.

In this review, translational evaluation is considered across three sequential phases: Phase 1, analytical feasibility; Phase 2, clinical validity; and Phase 3, clinical utility. Analytical feasibility requires reproducible recovery and measurement of the proposed EV signature in clinical biofluids. Clinical validity requires blinded external validation in independent multicenter cohorts using predefined thresholds, with assessment of sensitivity, specificity, and calibration. Clinical utility further requires comparison with standard clinical predictors and demonstration of incremental value over established liquid-biopsy analytes such as circulating tumor DNA (ctDNA) and CTCs. Most current BCSC-associated EV studies remain at the feasibility or preclinical stage, whereas prospective evidence demonstrating clinical utility remains limited.

### 4.1. Clinical Feasibility and Emerging Evidence

#### 4.1.1. Breast Cancer EV Detection

Liquid biopsy enables minimally invasive and repeated assessment of tumor-related materials in blood and other body fluids, allowing temporal changes in tumor biology to be monitored beyond the spatial and temporal limitations of tissue biopsy. EVs are attractive liquid-biopsy analytes because they carry protected proteins, nucleic acids, lipids, and membrane-associated molecules released by viable tumor and microenvironmental cells. Clinical studies have shown that enrichment of breast-cancer-associated EVs can improve the detection of disease-related miRNA and protein signatures compared with analysis of total circulating EV populations [94,95]. These findings support the feasibility of EV-based breast cancer diagnosis, but most existing signatures predominantly reflect tumor presence, overall disease burden, molecular subtype, or metastatic status.

The proposed diagnostic value of BCSC-associated EVs is more specific. Whereas EVs released by the broader breast cancer cell population primarily provide an averaged representation of tumor-derived molecular signals, EV populations associated with BCSCs may preferentially reflect cellular programs involved in self-renewal, phenotypic plasticity, treatment tolerance, dormancy, and metastatic niche formation. The biological basis for this concept is derived primarily from controlled experimental models. Experimental studies have shown that EVs from breast cancer stem-like populations carry bioactive cargos distinct from those of differentiated tumor cells and induce different stromal and metastatic responses [84]. EVs from BCSC-enriched donor populations have also been reported to promote epithelial–mesenchymal transition and metastatic progression through miRNA transfer [11]. Their potential diagnostic advantage is therefore not simply greater sensitivity for detecting breast cancer, but the possibility of identifying biologically aggressive cellular states within tumors of otherwise similar size, stage, or molecular subtype. In this sense, BCSC-associated EVs may function as state-resolved biomarkers of the residual populations most relevant to treatment failure and recurrence. However, findings obtained from cell-line or other controlled experimental systems cannot establish that corresponding circulating EV populations originate from functionally verified BCSCs in patients.

Circulating EVs have already shown potential for early breast cancer detection. Developmental endothelial locus-1 on circulating EVs differentiated patients with early breast cancer from control participants [99], while plasma and serum EV profiling identified diagnostic miRNA and protein panels associated with breast cancer and distant metastasis [95,100]. Immunoaffinity enrichment of breast-cancer-derived EVs produced a five-miRNA signature that performed better than total plasma EV analysis and complemented mammography, including in women with dense breasts [94]. Multiplex platforms such as MESS2CAN have further enabled simultaneous detection of HER2, EpCAM, and EGFR on EVs using small plasma volumes [13]. These studies demonstrate the diagnostic potential of tumor-enriched EVs, although direct evidence that a circulating signature specifically identifies BCSC states remains limited. Accordingly, current plasma- and serum-based findings should generally be interpreted as BCSC-associated EV signals or signatures unless their origin from functionally verified BCSCs has been independently established. No current platform has demonstrated direct recovery of EVs from functionally verified BCSCs in untreated whole blood. The next step is therefore to determine whether the addition of stemness- and resistance-related features can distinguish biologically high-risk disease beyond conventional tumor-associated EV detection.

Bioengineering technologies are essential for converting this biological rationale into a clinically applicable assay. Immunoaffinity enrichment can separate tumor-associated EVs from the large background of host-derived particles, while multiplex biosensors and single-EV platforms can determine whether tumor-associated surface molecules, stemness-related features, and functional cargos coexist within the same vesicle population. A single-molecule array assay has enabled rapid quantification of circulating EVs in early breast cancer [101], MESS2CAN has demonstrated multiplex surface profiling from approximately 10 μL of plasma [13], and single-vesicle nanoscopy has resolved molecular heterogeneity among HER2-enriched EVs [14]. These technologies support the development of BCSC-associated EVs as dynamic, state-resolved liquid-biopsy biomarkers. At present, however, they primarily establish analytical feasibility rather than clinical validity or utility for BCSC-associated EV detection.

#### 4.1.2. Treatment Monitoring

The distinctive clinical value of BCSC-associated EVs may be greatest in longitudinal assessment rather than in a single diagnostic measurement. Stem-like tumor states may persist at low abundance after treatment, emerge through cellular plasticity, or expand under therapeutic selection. Chemotherapy-exposed breast cancer cells can release EVs that transfer miRNAs and induce stem-like, treatment-resistant phenotypes in recipient cells [10]. Trastuzumab-resistant breast cancer cells likewise release EV populations with altered HER2 and tetraspanin characteristics, which can be resolved at the single-vesicle level and detected in patient plasma [14]. Serial analysis of EV populations carrying both tumor-associated and stemness-related features may therefore help identify treatment adaptation and residual resistant disease during or after therapy.

BCSC-associated EVs provide a different diagnostic dimension from CTCs. CTCs preserve intact cellular morphology and permit direct measurement of epithelial, mesenchymal, and stemness-associated phenotypes; stem-like CTC populations have been detected in patients with early breast cancer [102]. However, CTCs are rare, may be intermittently released, and principally represent cells that have entered the circulation. EVs are more abundant, require smaller sample volumes, and can be sampled repeatedly for surface proteins and internal molecular cargo. Importantly, tumor cells remaining within the primary tumor, bone marrow, or metastatic niche may release EVs without themselves appearing as detectable CTCs. BCSC-associated EVs may therefore offer broader surveillance of tissue-resident stem-like and treatment-adapted populations, whereas CTCs retain the advantage of direct intact-cell phenotyping and functional analysis. The two analytes should consequently be regarded as complementary rather than interchangeable. Whether BCSC-associated EV measurements provide incremental value over CTC-based monitoring requires prospective longitudinal validation.

#### 4.1.3. Recurrence Surveillance

The same capacity for repeated sampling may support recurrence surveillance, particularly if changes in EV-associated stemness or resistance features precede measurable tumor regrowth. Such signals could potentially identify recurrence-prone residual disease, but this application remains an emerging hypothesis rather than an established clinical use. The distinction from ctDNA is equally important. ctDNA provides highly informative tumor-genomic data, including somatic mutations, copy-number alterations, clonal evolution, and molecular residual disease. Longitudinal ctDNA analysis can identify patients at high risk of relapse and detect molecular recurrence before clinical progression [103], although sensitivity and lead time depend strongly on assay design [104]. BCSC-related phenotypes, however, are not determined exclusively by stable genomic alterations. Cellular plasticity, epigenetic regulation, transcriptional reprogramming, metabolic adaptation, quiescence, and microenvironmental signaling can generate treatment-tolerant states without producing a corresponding mutation detectable by ctDNA. Because EVs carry membrane proteins, regulatory RNAs, lipids, and other products of viable and actively adapting cells, BCSC-associated EVs may complement ctDNA by revealing functional and non-genetic dimensions of resistance.

Their most plausible clinical role is therefore within an integrated liquid-biopsy panel: ctDNA defines genomic residual disease and clonal evolution, CTCs characterize rare intact circulating cells, and BCSC-associated EVs may provide dynamic information on stemness, treatment adaptation, and recurrence-driving cellular states.

Their principal diagnostic value is unlikely to be the replacement of conventional breast cancer EV assays, CTCs, or ctDNA, but the identification and longitudinal monitoring of stem-like, treatment-resistant, and recurrence-prone tumor states that are incompletely represented by the other circulating analytes. Their incremental prognostic value should therefore be tested prospectively against established clinical variables and existing liquid-biopsy markers.

### 4.2. Preclinical Therapeutic Evidence

#### 4.2.1. Engineered EVs

Breast cancer stem cells (BCSCs) are difficult to eradicate because they combine drug efflux, enhanced DNA damage repair, redox and metabolic adaptation, anti-apoptotic signaling, quiescence, and protection from the TME [2]. Engineered EVs provide two complementary therapeutic strategies. EVs can be used as carriers to deliver chemotherapeutic agents, nucleic acids, proteins, or combination cargos to treatment-resistant tumor states. Alternatively, EV-mediated communication can itself be disrupted by inhibiting vesicle generation, release, uptake, or the activity of resistance-promoting cargos. The therapeutic rationale therefore extends beyond using EVs as passive nanocarriers and includes exploiting or interrupting their natural role in communication between BCSCs, differentiated tumor cells, and the surrounding microenvironment.

An important distinction should be made between EVs engineered to target BCSCs and EVs produced directly by BCSCs. BCSC- or breast-cancer-cell-derived EVs may retain membrane proteins, adhesion molecules, and other biological features that promote homotypic recognition and uptake by related tumor cells. This property could potentially improve delivery to residual stem-like tumor populations. However, the same vesicles may contain endogenous miRNAs, proteins, lipids, and signaling molecules that promote self-renewal, immune suppression, angiogenesis, drug resistance, or metastasis. Their composition may also vary according to tumor subtype, treatment exposure, and donor-cell state. Direct use of EVs derived from functionally validated BCSCs as therapeutic products therefore presents a greater biological risk than the use of well-characterized non-malignant donor cells. Mesenchymal stromal cells, dendritic cells, and other engineered producer cells are generally more suitable sources, while tumor-derived membranes may be considered only when their targeting advantage can be retained without transferring pathogenic cargo [105].

The choice of an engineered EV platform should also be justified against more mature nanocarriers, particularly liposomes and lipid nanoparticles (LNPs). Compared with synthetic nanocarriers, EVs possess naturally assembled lipid membranes containing proteins, glycans, receptors, and adhesion molecules that may facilitate cellular recognition, membrane interaction, intracellular trafficking, and, in selected settings, homotypic tumor-cell uptake. They can carry several classes of therapeutic cargo simultaneously and may protect proteins and regulatory RNAs within a biologically adapted membrane environment. EVs may consequently be advantageous when the treatment requires delivery of fragile biological molecules, engagement with specific cellular uptake pathways, or access to treatment-resistant cells that are poorly reached by conventional formulations [105,106]. For BCSC targeting specifically, the most distinctive potential advantage is the ability to combine a biologically active membrane interface with surface ligands and molecular cargos directed against stemness and resistance pathways. A recent *Bioengineering* review of siRNA delivery strategies for triple-negative breast cancer emphasizes benchmarking biological carriers against established lipid, polymeric and ligand-directed systems [107]. Target-journal research on reduced-graphene-oxide/silver nanocomposites has also incorporated direct BCSC phenotyping and functional endpoints, providing a relevant comparator for evaluating stem-cell-directed engineered carriers [108].

These biological advantages are counterbalanced by substantial pharmaceutical limitations. EV composition depends on the donor cell and culture conditions, resulting in greater heterogeneity and batch variability than generally encountered with synthetic nanoparticles. EV yields are often low, cargo encapsulation can be inefficient, isolation and purification are technically demanding, and particle number alone may not reliably predict biological potency. Biodistribution is also frequently dominated by uptake in the liver, spleen, and mononuclear phagocyte system. The use of tumor- or BCSC-derived EVs introduces the additional risk of transferring endogenous oncogenic or prometastatic signals. By contrast, liposomes and LNPs have more precisely defined compositions, tunable physicochemical properties, comparatively high and reproducible drug-loading capacity, established scale-up processes, and substantially greater pharmaceutical and clinical maturity [106,109]. Polymeric nanoparticles offer controlled degradation and drug release, while inorganic nanoparticles provide magnetic, optical, or thermal functions, although material persistence and toxicity may limit repeated administration. Hybrid platforms may partly reconcile these trade-offs by combining the biological membrane properties of EVs with the loading efficiency and formulation control of synthetic nanocarriers [110]. Exosome–liposome hybrids have been used to improve nucleic-acid loading [111], while exosome-coated porous silica nanoparticles have enabled combined delivery against EMT- and cancer-stem-cell-related phenotypes in triple-negative breast cancer [112].

Accordingly, EVs should not be selected for BCSC therapy solely because they are naturally derived. Their use is most justified when native membrane composition, homotypic recognition, intracellular trafficking, or protection of complex biological cargo produces a measurable advantage over a matched liposome, LNP, or other nanocarrier. A further potential advantage may arise in long-term or repeated administration, but only when engineered EVs demonstrate lower toxicity, inflammatory responses, or treatment-related intolerance than matched synthetic carriers. Direct administration of BCSC-derived EVs is unlikely to be the preferred strategy unless their pathogenic endogenous contents can be removed and their safety demonstrated. A more rational approach may involve non-malignant donor EVs engineered with BCSC-directed ligands, purified tumor-membrane components used as targeting templates, or EV–synthetic nanoparticle hybrids that preserve biological recognition while improving loading, reproducibility, and manufacturability. Clinical translation will ultimately require direct head-to-head comparisons of BCSC depletion, tumor recurrence, biodistribution, repeated-dose safety, production consistency, and therapeutic benefit rather than comparisons based only on particle uptake or short-term tumor shrinkage.

#### 4.2.2. BCSC Targeting

Surface engineering can improve the recognition and uptake of therapeutic EVs by displaying receptor-binding proteins, antibody fragments, peptides, aptamers, or lipid-conjugated ligands. Potential targets include CD44, EpCAM, HER2, integrins, and other receptors enriched in selected breast cancer or treatment-resistant states, although none is universally restricted to BCSCs. In HER2-positive breast cancer, EVs displaying a HER2-binding designed ankyrin repeat protein delivered TPD52 siRNA and reduced target-gene expression [113]. HER2-targeted EVs loaded with doxorubicin also showed preferential interaction with HER2-positive cells and inhibited tumor growth [114]. In doxorubicin-resistant triple-negative breast cancer, CL4-aptamer-modified EVs delivered DARS-AS1 siRNA and restored drug sensitivity [115]. These studies demonstrate the feasibility of receptor-directed EV delivery, but most measured general tumor-cell cytotoxicity or tumor-volume reduction. A BCSC-directed effect is more convincingly supported when treatment also reduces stem-like cell fractions, serial sphere-forming capacity, resistance after drug withdrawal, or tumor-initiating potential. An engineered decoy-EV system that co-targeted TEAD4 signaling and mitochondrial vulnerability in triple-negative breast cancer provides a recent example in which cancer-stem-cell effects were incorporated into a multilevel therapeutic design [116].

BCSC targeting can also involve disruption of EV-mediated signaling that maintains stemness or treatment resistance. Rab27-dependent trafficking contributes to EV docking and release, and Rab27a inhibition reduced primary tumor growth and lung dissemination in a metastatic breast cancer model [117]. Pharmacological inhibition of neutral sphingomyelinase with GW4869 reduced EV-mediated intercellular mitochondrial transfer and attenuated acquired chemoresistance in triple-negative breast cancer [118]. More selective strategies may target specific resistance-associated cargos or their downstream pathways. EV-associated miR-9-5p promoted tamoxifen resistance and stemness-related changes in recipient breast cancer cells [85], whereas EV-associated Linc00969 enhanced trastuzumab resistance through HER2 regulation and autophagy [119]. Targeting specific vesicular cargos or recipient-cell signaling may therefore provide greater selectivity than globally suppressing EV production, which could also interfere with physiological communication among normal cells.

#### 4.2.3. Drug Delivery

Therapeutic cargos can be incorporated during EV production through donor-cell transfection, genetic engineering, or drug exposure, or after EV isolation through incubation, electroporation, sonication, extrusion, membrane permeabilization, and related methods [105]. Cargo selection may include cytotoxic drugs, siRNAs, miRNA mimics or inhibitors, antisense oligonucleotides, proteins, and combinations intended to induce tumor-cell death while suppressing self-renewal or drug-resistance pathways. A dendritic-cell-derived exosome platform loaded with doxorubicin showed uptake by breast cancer cells and stem-like populations, reduced cancer-stem-cell-associated markers in murine tumors, and produced limited cytotoxicity in normal mammary cells and peripheral blood mononuclear cells [120]. Engineered EVs have also been used to co-deliver chemotherapeutic agents and survivin-directed siRNA in triple-negative breast cancer models [121]. Nevertheless, loading efficiency remains cargo- and method-dependent, and active-loading procedures may induce cargo aggregation, membrane disruption, or loss of vesicle activity. The biological effect should therefore be assessed according to the amount of functional cargo delivered rather than the nominal amount initially added during loading.

### 4.3. Exploratory Future Applications

#### 4.3.1. EV Vaccines

EV-based cancer vaccines can be developed by using tumor-derived EVs as antigen sources, dendritic-cell-derived EVs as antigen-presenting platforms, or engineered EVs displaying defined tumor antigens and immune stimulators. A MUC1 glycopeptide conjugated to dendritic-cell-derived exosomes induced antigen-specific humoral and cytotoxic T-cell responses and inhibited MUC1-positive tumors in preclinical models [122]. Dendritic-cell-derived EVs may also amplify neoantigen-specific immunity by transferring antigen-presentation and costimulatory signals to recipient monocytes [123]. However, these studies do not establish selective immunity against BCSCs. An approach should be described as a BCSC-targeted vaccine only when the antigen is enriched in functionally verified BCSCs and vaccination depletes tumor-initiating cells. Tumor-derived EVs additionally require caution because they may contain immunosuppressive or prometastatic cargo.

#### 4.3.2. Resistance Screening

EVs may also be incorporated into resistance-screening and companion-diagnostic models. Patient-derived organoids can reproduce individual responses to neoadjuvant breast cancer therapy [90], while circulating EV profiles have been explored for treatment-response prediction [76]. A potential workflow would expose matched organoids or tumor-on-chip models to patient EVs collected before and during treatment and determine whether EV-induced changes in viability, stemness, or drug sensitivity anticipate the clinical response. Such a model would require standardized longitudinal sampling, EV-depleted controls, mechanistic cargo perturbation, and blinded comparison with pathological or radiological outcomes. At present, these approaches remain experimental because EV-dependent responses in ex vivo models have not been prospectively established as predictors of patient treatment response.

#### 4.3.3. Companion Diagnostics

A related future application is the development of BCSC-associated EV signatures as companion diagnostics for treatment stratification. Longitudinal EV profiles could potentially identify treatment-adapted or resistant cellular states and thereby support selection or modification of therapy. However, evidence that such EV signatures improve treatment selection beyond established clinical and molecular predictors is currently insufficient. Their development as companion diagnostics would therefore require predefined biomarker thresholds, independent prospective validation, and demonstration that EV-guided treatment decisions provide clinically meaningful incremental value.

EV vaccines, resistance-screening systems, and companion-diagnostic applications should therefore be regarded as exploratory directions until BCSC specificity, reproducibility, safety, and prospective clinical utility are established.

## 5. Challenges and Future Perspectives

### 5.1. Biological and Analytical Challenges

A central challenge in BCSC research is that stemness is neither confined to a single phenotypic population nor stable across disease contexts. The abundance, marker expression, and molecular programs of BCSCs vary among breast cancer subtypes, primary and metastatic sites, treatment stages, and microenvironmental conditions [18,124]. Differentiated tumor cells may also acquire stem-like properties in response to inflammatory signaling or therapeutic stress, thereby continuously reshaping the BCSC compartment [3,125]. Findings obtained from established cell lines, mammospheres, patient-derived tumors, organoids, circulating tumor cells, and metastatic specimens therefore cannot be assumed to represent equivalent biological states. This heterogeneity also complicates the interpretation of BCSC-related EVs because changes in vesicle composition may arise from intrinsic stemness, subtype-specific signaling, treatment adaptation, or broader cellular stress. This heterogeneity remains a major challenge for source-resolved interpretation of BCSC-associated EVs.

Analytical variability represents an additional barrier to detecting BCSC-associated EV signals in clinical samples. Circulating EVs originate from tumor cells, platelets, immune cells, endothelial cells, and other host tissues, whereas vesicles associated with rare stem-like tumor states are likely to account for only a small proportion of the total population. Plasma or serum selection, anticoagulant, processing delay, platelet depletion, hemolysis, storage, and freeze–thaw exposure can alter EV concentration and molecular composition [64,65]. Recent breast cancer biobanking and liquid-biopsy studies further indicate that plasma isolation procedures and collection-to-processing workflows affect sample quality, platelet contamination, and downstream EV measurements [126,127]. MISEV2023 provides a general framework for EV nomenclature, separation, characterization, and functional interpretation, but interlaboratory comparability remains limited [15]. Instrument calibration and standardized fluorescence or light-scatter units can improve cross-platform comparison, while single-vesicle assays require controls for detection sensitivity, coincidence, and molecular colocalization [77,78]. Progress will therefore depend on standardized workflows and validation across independent clinical cohorts.

### 5.2. Manufacturing, Safety, and Regulatory Challenges

Therapeutic translation requires EVs to be developed as reproducible biological products rather than laboratory-scale particle preparations. Donor-cell source, passage, genetic stability, culture medium, oxygen tension, bioreactor conditions, stimulation, and harvest interval can all alter EV yield and cargo. Downstream processing must balance recovery with the removal of soluble proteins, lipoproteins, nucleic-acid complexes, residual media components, and process-related impurities. Comparative studies have shown that isolation workflows can produce substantial differences in EV recovery, reproducibility, molecular composition, and functional readouts [66,67]. Scalable manufacturing studies nevertheless demonstrate that controlled cell expansion and EV purification can be achieved under increasingly standardized conditions [128]. Product characterization should connect particle identity and purity with cargo loading, membrane integrity, storage stability, and mechanism-related biological activity. Particle concentration alone is insufficient because preparations containing similar particle numbers can differ substantially in potency [129]. For BCSC-targeted products, the potency assay should reflect the intended mechanism, such as depletion of stem-like populations, suppression of self-renewal, reversal of treatment resistance, or reduction in tumor-initiating capacity. Bioengineering data showing that three-dimensional matrix composition, oxygen tension and dynamic culture alter EV production further support treating culture conditions as controlled manufacturing variables rather than background details [130].

Safety evaluation must consider both the engineered therapeutic cargo and the endogenous biological activity of the EV carrier. Systemically administered EVs frequently accumulate in the liver, spleen, lungs, and mononuclear phagocyte system, potentially reducing tumor delivery and exposing non-target tissues. Repeated administration may additionally affect immune responses, complement activation, coagulation, and organ function. Tissue factor and other procoagulant components represent potential concerns for selected cell-derived EV products [131]. Tumor-derived EVs, including EVs from functionally validated BCSCs, introduce an additional risk because residual endogenous cargos may support survival, stemness, immune suppression, angiogenesis, or metastasis. Therapeutic development should therefore generally favor qualified non-malignant donor cells unless a tumor-derived membrane feature provides a clear and controllable targeting advantage [105]. Donor source, cargo type, surface modification, and degree of manipulation may also influence regulatory classification and the required nonclinical program. Current translational frameworks emphasize donor-cell qualification, manufacturing consistency, impurity control, biodistribution, repeated-dose toxicology, tumor-promoting risk, and mechanism-related potency testing [132]. These requirements are especially important because liposomes and lipid nanoparticles already provide more mature benchmarks for formulation control, scale-up, storage, and pharmaceutical quality.

### 5.3. Future Perspectives: Integrated Bioengineering and Precision Translation

Future progress will depend on integrating technologies that are currently applied as separate experimental steps. A sample-to-answer platform could combine tissue dissociation, removal of dead and non-target cells, BCSC enrichment, single-cell phenotyping, standardized EV collection, and multiparameter single-EV analysis within one coordinated workflow. Microfluidic systems have already linked breast cancer spheroid culture with downstream EV harvesting and single-vesicle analysis [97], while droplet-array platforms have enabled measurement of EV secretion from individual breast cancer cells during targeted treatment [57]. Patient-derived breast cancer organoids retain important histological and molecular characteristics of their source tumors and can reproduce interpatient variation in therapeutic response [89,90]. Organoid microarrays may further improve throughput and experimental consistency [91]. Combining these systems with perfused tumor-on-chip models could reveal how specific BCSC states influence EV output and how the resulting vesicles remodel stromal, vascular, immune, and metastatic compartments. Breast-cancer-derived EVs have already been studied in vascularized organ-specific chips, where they altered endothelial-barrier properties and subsequent tumor-cell adhesion [82]. Multicellular models have also demonstrated that EVs from stem-like and differentiated breast cancer populations can produce distinct fibroblast, endothelial, and metastatic-niche responses [84]. These platforms may therefore connect cellular identity, EV phenotype, and biological function more directly than studies based on mixed donor populations or bulk circulating EVs.

Computational and clinical integration represents a second major future direction. Artificial intelligence may support automated classification of cells and EVs, interpretation of imaging and spectroscopic signals, integration of proteomic and RNA features, and longitudinal prediction of treatment response or recurrence. AI-assisted surface-enhanced Raman spectroscopy has already been used to classify serum EV patterns in breast cancer, illustrating the potential of computationally enhanced, label-free detection [75]. Its clinical utility, however, will depend on biologically meaningful reference labels, control of batch and center effects, independent validation, and interpretable relationships with clinical outcomes. Multianalyte liquid biopsy is likely to be particularly valuable: ctDNA can define genomic residual disease and clonal evolution, CTCs can characterize rare intact circulating cells, and BCSC-associated EVs can report information on viable-cell signaling, non-genetic plasticity, and treatment-adaptive states [94,102,103]. Near-term development should prioritize longitudinal treatment-response and recurrence monitoring, where within-patient changes may be more informative than universal population thresholds. Diagnostic applications are also likely to mature earlier than systemically administered EV therapeutics because they do not require large-scale production of a complex biological product. For therapy, promising directions include non-malignant donor EVs carrying BCSC-directed ligands, selective inhibition of pathogenic EV signaling, and EV–synthetic nanoparticle hybrids that combine biological recognition with greater loading and manufacturing control. Across both diagnostic and therapeutic applications, future studies must move beyond technical feasibility and demonstrate incremental value in clinical risk stratification, treatment selection, recurrence prevention, or patient outcomes.

### 5.4. Limitations of the Current Review

This review has several limitations. First, as an engineering-focused narrative review, it does not provide a quantitative meta-analysis of all technical performance parameters. Second, BCSC donor-cell identification and functional validation vary considerably across the published literature, creating potential bias in EV source attribution among some of the studies included in this review. Third, most BCSC-associated EV biomarkers still lack validation in large, prospective, multicenter clinical studies, limiting the available evidence for clinical validity and utility. Finally, differences in sample preparation, EV isolation, characterization, and detection protocols limit direct quantitative comparisons of performance across platforms.

## 6. Conclusions

Bioengineering technologies are advancing the isolation, characterization, and translational evaluation of BCSC-associated EVs. However, major gaps remain in standardized BCSC donor-cell validation, reliable EV source attribution, consistent analytical and functional evaluation, and evidence of clinical value beyond existing biomarkers. Future work should prioritize standardized source attribution through validated donor-cell and single-EV analysis, prospective evaluation of the added value of BCSC-associated EV signatures for minimal residual disease and recurrence monitoring, and scalable GMP-compatible engineering with direct comparisons against liposomes and lipid nanoparticles for efficacy and safety. Addressing these priorities is essential for establishing whether BCSC-associated EVs can provide clinically useful biomarkers or therapeutic platforms.

## Figures and Tables

**Table 1:** Comparison of BCSC isolation and sorting platforms discussed in the current manuscript.

Platform Category	Recognition or Separation Basis	Purity and Workflow Role	Recovery Yield	Cell Viability	Throughput	Stemness Preservation and Validation	Representative Quantified Benchmarks
Flow cytometry (FACS; multiparameter/spectral) [16,17,22,23,24,25]	CD44/CD24, ALDH activity, EpCAM, CD49f, CD133 and viability panels	High marker-defined purity after optimized gating; second-stage high-purity sorting	Moderate; rare-event losses and sample preparation reduce the recovered target-cell fraction	Moderate to high with low pressure, appropriate nozzle size, temperature control and rapid collection	Moderate to high; decreases for rare events and complex multiparameter panels	Usually compatible with downstream assays, but phenotype alone requires independent functional confirmation	NR
Magnetic-activated cell sorting (MACS) [26,27,28]	Positive/negative selection using antibody-functionalized magnetic particles	Moderate purity; first-stage pre-enrichment or depletion before higher-resolution sorting	Moderate to high; bead retention and release chemistry can reduce recovery	Generally high when labeling and release are gentle	High; suitable for large-volume or debris-rich inputs	Generally preserved, but magnetic capture does not establish self-renewal or tumor initiation	NR
Passive microfluidic sorting [34,35,36]	Size, inertia, Dean flow, deformability, adhesion and hydrodynamic behavior	Low to moderate purity; label-free first-stage pre-enrichment	Moderate to high; depends on clogging, cluster handling and overlap in physical phenotypes	Generally high, although constrictions and shear can damage sensitive cells	High in continuous-flow designs	Uncertain because EMT, treatment and cell-cycle state alter physical properties; marker and functional testing required	Purity: 90–95% [35]; throughput: 1–2 mL/h [34]
Acoustic microfluidic sorting [37,38]	Acoustic radiation forces and acoustic streaming	Moderate purity; active second-stage enrichment of viable tumor-cell populations	Moderate to high; device- and matrix-dependent	High when thermal exposure and acoustic conditions are controlled	Moderate to high	Direct BCSC preservation is insufficiently validated; post-sort functional confirmation required	MCF7 recovery: 72.9–77.3%; WBC depletion: 99.9% [38]
Dielectrophoretic and other active microfluidic sorting [39,40,41]	Frequency-dependent polarizability; magnetic, optical or impedance-assisted manipulation	Moderate to high after sample-specific optimization; active second-stage manipulation	Moderate; losses occur during capture and release	Moderate to high when conductivity, field strength and Joule heating are controlled	Low to moderate for complex or single-cell workflows	Physical or electrical phenotype must be linked to subtype-adapted markers and functional endpoints	Recovery: ~95% at 50 μL/h [39]; capture efficiency: 96% at 0.5 mL/h [41]
Antibody-based affinity capture [44,45]	Antibodies or peptides immobilized on beads, microposts, channels, hydrogels or nanostructures	High marker-defined purity; targeted molecular enrichment	Low to moderate; strong binding and release chemistry can limit cell recovery	Moderate to high with gentle, reversible release	Low to moderate; limited by binding kinetics and surface area	Capture may preserve viable cells but cannot by itself establish BCSC identity	Purity: 60%; capture yield: >65% at 1–2 mL/h [44]
Aptamer and nanostructured affinity platforms [46,47,48,49,50,51]	Synthetic aptamers, multivalent ligands and high-area nano/microinterfaces	Moderate to high; tunable multivalent capture of heterogeneous phenotypes	Moderate; improved by reversible or degradable interfaces	Moderate to high; substrate signaling and release conditions require control	Low to moderate	Post-release stemness must be tested because topography and stiffness may alter cell state	Capture efficiency: 71–83% [48]; 90.9 ± 9.2% [49]
Droplet, microwell and single-cell platforms [32,54,55,56,57]	Single-cell compartmentalization, imaging, barcoding and functional reporters	High analytical purity at the single-cell level; functional stratification rather than bulk enrichment	Low to moderate; cell retrieval and doublet control are design-dependent	Variable; depends on compartment chemistry, occupancy and recovery method	Low to moderate for recoverable-cell workflows	Can directly assess clonality and functional heterogeneity, but transcriptomic state alone is not definitive	Single-cell capture efficiency: 34% [55]

**Note:** The five engineering dimensions are defined consistently across platforms: purity refers to the target-cell fraction among recovered cells (recovered target cells/total recovered cells); recovery yield refers to the recovered fraction of input target cells; viability refers to post-processing live-cell status; throughput refers to sample or cell processing capacity (recovered target cells/input target cells (recovered target cells/input target cells); and stemness preservation refers to retention of functional stem-cell properties after processing. Ratings are qualitative or semi-quantitative and represent typical trends rather than absolute rankings because specimen type, target definition, denominator, device design and downstream endpoint differ among studies. Representative quantified benchmarks are taken directly from the cited studies and retain the reported experimental definitions, denominators, and conditions. Qualitative ratings (high/moderate/low) represent the authors’ engineering assessment and are presented separately from experimentally reported numerical values. NR, no directly comparable quantitative engineering metric reported in the cited studies.

**Table 2:** Comparison of engineering platforms for BCSC-related EV isolation, detection and characterization.

Platform	Principle	Typical Sample Context	Main Output	Main Advantages	Main Limitations	Intact-EV Single-EV Capability	Representative Quantified Benchmarks
**A. Separation and enrichment**							
Differential ultracentrifugation and density gradients [15,66,67]	Sequential high-g pelleting and/or buoyant-density separation	Conditioned medium; plasma/serum	Broad EV-enriched fractions	Widely available; density gradients improve separation from soluble material	Co-pelleting, aggregation, rotor dependence, long processing and sample loss	Intact EVs usually recovered; not intrinsically single-EV	EV yield: 18.6–21.5 × 10^8^ particles/mL plasma (sUC) [66]
Size-exclusion chromatography and ultrafiltration [66,67]	Hydrodynamic size separation and membrane concentration	Plasma/serum; conditioned medium	Broad EV fractions or concentrated samples	Gentle, reproducible and compatible with functional studies	Lipoprotein overlap, dilution, adsorption, membrane-dependent loss and clogging	Intact EVs generally retained; not intrinsically single-EV	Particle-concentration enrichment: 266 ± 122-fold (serum); 1325 ± 376-fold (plasma) vs. UC [67]
Polymer precipitation [15,67]	Reduction in particle solubility	Plasma/serum; conditioned medium	High apparent particle recovery	Simple and scalable	Substantial co-precipitation of proteins, lipoproteins and non-vesicular material	Variable intact recovery; no single-EV resolution	NR
Immunoaffinity capture [70,71]	Antibody/aptamer binding to selected EV surface markers	Plasma/serum; conditioned medium	Marker-defined EV subpopulations	High enrichment for a chosen epitope; compatible with multiplex analysis	Biased subset; epitope dependence; release can be difficult	Possible with cleavable release; single-EV readout depends on downstream system	Capture efficiency: 82%; isolation time: 10 min [70]
Integrated microfluidic isolation [68,70,71]	Size, affinity, centrifugal, acoustic or electrokinetic modules	Low-volume plasma/serum or conditioned medium	Rapid isolation, concentration and sometimes direct detection	Low sample volume; automation; reduced transfer loss; modular integration	Cartridge variability, clogging, matrix effects and incomplete cross-platform standardization	Intact release possible; can be coupled to single-EV analysis	Separation efficiency: 90%; extraction purity: 85%; processing time: 8 min [68]
**B. Detection and characterization**							
Surface plasmon resonance and optical biosensing [74]	Refractive-index or optical signal change after EV binding	Plasma after preparation; isolated EVs	Real-time detection of surface-marker-defined EVs	Label-free or low-label detection; rapid and potentially quantitative	Surface fouling, matrix effects and calibration dependence	Usually ensemble measurement; intact particles remain captured	LOD: 2.1 × 10^7^ particles/mL (buffer); 1.1 × 10^8^–7 × 10^8^ particles/mL (plasma) [74]
Electrochemical biosensing [81]	Current, impedance or potential changes after EV/cargo recognition	Biofluids or isolated EVs	Compact protein or nucleic-acid readout	Sensitive, miniaturizable and compatible with point-of-care formats	Electrode fouling, antibody orientation and matrix interference	Usually bulk/ensemble; intact recovery is uncommon	NR
Raman and surface-enhanced Raman spectroscopy [75,76]	Molecular vibrational fingerprints with or without signal enhancement	Serum/plasma EV preparations	Bulk spectral profiles or tagged molecular signatures	Rich multiplex information and label-free classification potential	Batch effects, preprocessing dependence and risk of model overfitting	Potential single-particle analysis, but most clinical studies remain ensemble-based	Prediction accuracy: 100% [75]; AUC > 0.89 and accuracy > 0.94 [76]
High-resolution flow cytometry and imaging [14,77,78]	Calibrated light scatter and fluorescence or multiparametric imaging	Isolated EV preparations	Surface-marker co-expression and EV heterogeneity	Multiparameter single-EV counting and visualization	Swarm detection, thresholding, refractive-index and calibration challenges	Yes, when instrument sensitivity and controls are adequate	NR
Digital and single-EV molecular analysis [14,79]	Microwells, digital immunoassays or coupled surface-protein/cargo detection	Isolated EVs; plasma/serum	Rare EV subpopulations and protein–RNA co-localization	High analytical sensitivity and direct molecular co-occurrence information	Assay complexity, incomplete standards and limited independent cohorts	Designed for single-EV analysis; typically analytical rather than preparative	Detection limit: 214 EVs/μL [79]; localization precision: ~7–8 nm [14]

**Note:** Platform performance depends on sample matrix, pre-analytical handling and downstream objective. Tumor-associated surface-marker capture improves enrichment but does not alone establish BCSC origin. Single-EV capability indicates whether individual particles and marker co-localization can be analyzed; it does not imply preparative recovery. Purity is evaluated according to the denominator reported in the cited study, including particle-to-protein ratios (particles/µg protein by NTA/BCA) or particle-to-particle measures (target marker-positive particles/total particles), where applicable. Recovery/yield is defined as recovered target EVs relative to the initial input in raw biofluids or spiked controls, where reported. Representative quantified benchmarks retain the reported analytical denominators. NR, no directly comparable quantitative engineering metric reported.

**Table 3:** Representative biological and translational studies of BCSC-associated and breast-cancer-related extracellular vesicles.

Study	Model/Evidence Level	BCSC Validation	Key EV Cargo/Feature	Major Finding	Translational Relevance/Limitation
Kim et al., 2020 [82]	Breast cancer EVs; 3D human liver-chip	None	EV-associated TGF-β1	Breast cancer EVs induced endothelial-to-mesenchymal transition and barrier disruption in liver sinusoidal endothelial cells and increased fibronectin expression, facilitating breast cancer cell adhesion	Physiologically relevant model of EV-mediated metastatic-niche remodeling; not BCSC-specific
Sandiford et al., 2021 [93]	MSC–breast cancer cell interaction; in vitro/in vivo bone-marrow dormancy model	CSC-like transition assessed by molecular and functional features	MSC-derived EVs	MSC-derived EVs promoted stepwise dedifferentiation of breast cancer cells toward a dormant stem-like state in the bone-marrow perivascular niche	Supports microenvironmental EV regulation of breast cancer stem-like states; EVs are MSC-derived, not BCSC-derived
Liu et al., 2021 [85]	Tamoxifen-resistant breast cancer cells; in vitro/in vivo	None	miR-9-5p/ADIPOQ	Exosomal miR-9-5p transferred tamoxifen resistance to sensitive breast cancer cells through ADIPOQ downregulation	Links EV signaling to endocrine resistance; BCSC origin not established
Jafari et al., 2021 [9]	Primary human adipocyte-derived exosomes; breast cancer cell models	None	TSP5; BRD2/BRD4-associated signaling	Adipocyte-derived exosomes induced EMT- and CSC-associated gene expression in breast cancer cells, with stronger effects from T2D or insulin-resistant adipocytes	Supports adipocyte-to-tumor EV regulation of breast cancer stem-like traits; EVs are adipocyte-derived, not BCSC-derived
Li et al., 2022 [11]	BCSC-enriched MDA-MB-231 and SUM149PT models; in vitro/in vivo	BCSC donor populations used; functional validation less stringent than proposed Tier 2/3 standard	miR-197/PPARG	BCSC-EVs promoted EMT, tumor growth, and liver metastasis through miR-197-mediated PPARG suppression	Direct BCSC-EV mechanistic evidence; cell-line-derived BCSC model without clinical source validation
González-Callejo et al., 2023 [84]	CSC versus differentiated MDA-MB-231 TNBC cells; in vitro/in vivo	CSC and differentiated populations selectively isolated and characterized	Distinct EV cargo profiles	CSC-EVs activated myofibroblastic CAFs, enhanced endothelial remodeling, and established a lung premetastatic niche	Strong source-resolved evidence for CSC-EV–TME crosstalk; preclinical evidence
Santos et al., 2024 [87]	Drug-resistant breast cancer cells; macrophage models	None	Resistance-associated EV profiles	Resistant-cell EVs promoted acquired drug resistance and pro-inflammatory macrophage responses	Connects EV-mediated resistance with immune remodeling; BCSC origin not established
Kim et al., 2024 [94]	Clinical plasma from breast cancer patients and controls	None; tumor-associated EV enrichment	Five-miRNA EV signature	Tumor-enriched EV miRNA profiling improved diagnostic performance over total-EV analysis	Clinical evidence for tumor-enriched EV biomarkers; signature is not BCSC-specific
Xu et al., 2024 [95]	Clinical serum EVs	None	EV protein signatures	Serum-EV proteomics identified diagnostic and metastatic breast cancer signatures and TALDO1 as a distant metastasis-associated EV protein	Supports clinical EV biomarker development; broad circulating EVs with unresolved BCSC origin
Jiang et al., 2024 [14]	Breast cancer models and patient plasma	None	HER2 and multiparametric EV features	Single-EV nanoscopy resolved molecular heterogeneity among HER2-enriched EVs	Enables high-resolution analysis of clinically relevant EV subpopulations; HER2 enrichment does not establish BCSC origin
Song et al., 2025 [88]	Resistant breast cancer cell models	None	BANCR	BANCR-containing EVs promoted trastuzumab resistance through the miR-34a-5p/HMGA1/Wnt/β-catenin axis	Mechanistic evidence for EV-mediated trastuzumab resistance; BCSC origin not established
Jia et al., 2025 [76]	HER2-positive breast cancer patients receiving neoadjuvant therapy	None	Raman EV signatures	EV spectral profiles combined with machine learning were evaluated for treatment-response prediction	Potential longitudinal treatment-monitoring strategy; BCSC source attribution and prospective validation lacking
Fan et al., 2026 [96]	Treatment-naïve TNBC specimens and preclinical models	CSC populations profiled using single-cell approaches	TSPAN8+ CSC-derived EVs	TSPAN8+ CSC-EVs promoted CD103+FOXP3+ Treg formation through CD103/LKB1–AMPK–FOXP3 signaling; EV-TSPAN8 blockade enhanced anti-PD-1 activity	Links CSC-EVs directly to immune suppression and identifies a therapeutic target; clinical therapeutic utility remains unproven
Rima et al., 2022 [97]	Breast cancer tumor spheroids; 3D microfluidic model	None	Single-EV tetraspanin profiles	Microfluidic culture enabled low-volume harvesting of spheroid-derived EVs and analysis of biomarker expression and tetraspanin coexpression at the single-EV level	Bioengineering platform for source-controlled EV production and single-EV analysis; not BCSC-specific
Guo et al., 2021 [98]	Paclitaxel-resistant and paclitaxel-sensitive breast cancer cells; in vitro	BCSC enrichment demonstrated in paclitaxel-resistant breast cancer cells	ANXA6-containing exosomes	ANXA6-containing exosomes promoted paclitaxel resistance, autophagy, growth, migration, and stemness through YAP1-associated signaling	Directly links BCSC-enriched breast cancer cells and EV-mediated chemoresistance; preclinical evidence without clinical validation

## Data Availability

No new data were created or analyzed in this study. Data sharing is not applicable to this article.
